# Supplementary material for: Hyperbaric oxygen therapy in alleviating cerebral ischemia-reperfusion injury via the BMP6/Smad-hepcidin pathway
Source: PLoS One. 2026 Jan 12;21(1):e0339455. doi: 10.1371/journal.pone.0339455 (PMC12795386; doi:10.1371/journal.pone.0339455)
Supplement: S1 File — (DOCX) [file pone.0339455.s001.docx]

Supplementary Methods

1. Main Experimental Reagents and Instruments

BMP6 Antibody: Purchased from Abcam.

SMAD2 Antibody: Purchased from Cell Signaling Technology.

QuickBlock™ Western Antibody Dilution Buffer: Purchased from Shanghai Beyotime Biotechnology Co., Ltd.

GAPDH Antibody: Purchased from Shanghai Tuoran Biotechnology Co., Ltd.

Cleaved Caspase-3 (Asp175) Antibody (100 µL): Purchased from Cell Signaling Technology.

SMAD4 Antibody: Purchased from Proteintech.

Isoflurane (anesthetic): Purchased from Shenzhen Reward Life Technology Co., Ltd.

PAGE Gel Preparation Kit (10%): Purchased from Vazyme Biotech Co., Ltd.

Ferroportin (FPN1) Antibody: Purchased from LeiYi Medical, targeting membrane iron transporter protein 1.

GPX4 Antibody: Purchased from LeiYi Medical.

Skim Milk: Purchased from Sangon Biotech (Shanghai) Co., Ltd.

Anti-Hepcidin-25 Antibody: Purchased from Abcam.

Hifair® III First Strand cDNA Synthesis SuperMix for qPCR (with gDNA digester): Purchased from Yeasen Biotechnology Co., Ltd.

Tissue RNA Purification Kit: Purchased from Yeasen Biotechnology Co., Ltd.

Taq SYBR Green qPCR Premix (Universal): Purchased from Yuanxiang Medical Equipment Co., Ltd.

Protein Marker: Purchased from Yuanxiang Medical Equipment Co., Ltd.

HRP-linked Secondary Antibodies: Anti-Rabbit IgG and Anti-Mouse IgG purchased from Cell Signaling Technology.

QuickBlock™ Western Primary Antibody Dilution Buffer: Purchased from Shanghai Beyotime Biotechnology Co., Ltd.

Chloral Hydrate: Purchased from Dongting Pharma (Changde), Hunan Province.

Electrophoresis and Transfer Systems: Bio-Rad electrophoresis systems were used.

Automated Sample Grinding Machine: Purchased from Jinxin.

Automated Chemiluminescence Imaging System: Tanon 4600, used for imaging Western blots.

Ultramicrotome: Leica UC7 used for ultrathin sectioning.

Transmission Electron Microscope: ThermoFisher Talos 120 for observing and imaging ultrastructural changes.

Primary Antibodies for Nrf2, GAPDH, and Secondary Antibodies: Purchased from Shanghai Shenggong Biotechnology Co., Ltd.

Oxidative Stress Markers (SOD, GSH-Px, MDA): Assay kits purchased from Shanghai Tongren Chemical Research Institute.

Chicken Anti-GFAP Antibody: Purchased from Merck Millipore, Germany.

BMP6/Smad Antibody: Purchased from Shanghai Baijian Biotechnology.

Recombinant BMP6: Purchased from PeproTech; Recombinant Protein BEMPER (Catalog No. 2299-CV-050) purchased from R&D Systems, USA.

Experimental Animals: Male Wistar rats (250-280 g, SPF grade) were purchased from Shanghai Slac Laboratory Animal Co., Ltd. The animals had free access to food and water and were allowed to acclimate for one week before the experiment.

2. Western Blotting/ELISA Detection of BMP6 Protein Changes

BMP6 Protein Immunoblotting Protocol: For Western blotting, membranes were incubated with primary antibodies diluted in 5% bovine serum albumin (BSA), 1X Tris-buffered saline (TBS), and 0.1% Tween® 20 at 4°C overnight with gentle shaking. The following day, membranes were washed and incubated with HRP-conjugated secondary antibodies. Enhanced chemiluminescence (ECL) was used for detection, and images were captured using a Tanon 4600 chemiluminescence system.

Western Blot Reagents:

PBS Buffer: 20X phosphate-buffered saline, diluted to 1X with deionized water.

TBS Buffer: 10X Tris-buffered saline, diluted to 1X with deionized water.

SDS Sample Buffer: Prepared fresh before each use by mixing 30X dithiothreitol (DTT) with 3X SDS sample buffer, then diluted to 1X.

Electrophoresis Buffer: 10X Tris-glycine-SDS buffer, diluted to 1X with deionized water.

Transfer Buffer: 10X Tris-glycine transfer buffer, diluted to 1X with methanol and water.

Blocking Solution: 5% skim milk in 1X TBST.

HRP-Conjugated Secondary Antibodies: Diluted in 1X TBST with 5% BSA.

Signal Detection: ECL reagents were prepared fresh and used for visualizing protein bands on X-ray films or digital imaging systems.

3. Western Blotting/ELISA Detection of Smad1 Protein Changes

Similar to the BMP6 protocol, the Smad1 protein detection followed Western blotting procedures, using appropriate dilutions for primary and secondary antibodies, and employing ECL for visualization.

1. qRT-PCR of Ferroportin

| Rat-actin-F | CCAACACAGTGCTGTCTGG |
| --- | --- |
| Rat-actin-R | GTGGACAGTGAGGCCAGG |
| Rat-Hepcidin-F1 | CGGCAACAGACGAGACAGAC |
| Rat-Hepcidin-R1 | GAGGCATATGGGGAAGTTGG |
| Rat-Hepcidin-F2 | GGCTGCCTGTCTCCTGCTTC |
| Rat-Hepcidin-R2 | GCGCACTGTCATCAGTCTTG |
