# Supplementary figures and images for: Hyperbaric oxygen therapy in alleviating cerebral ischemia-reperfusion injury via the BMP6/Smad-hepcidin pathway
Source: PLoS One. 2026 Jan 12;21(1):e0339455. doi: 10.1371/journal.pone.0339455 (PMC12795386; doi:10.1371/journal.pone.0339455)

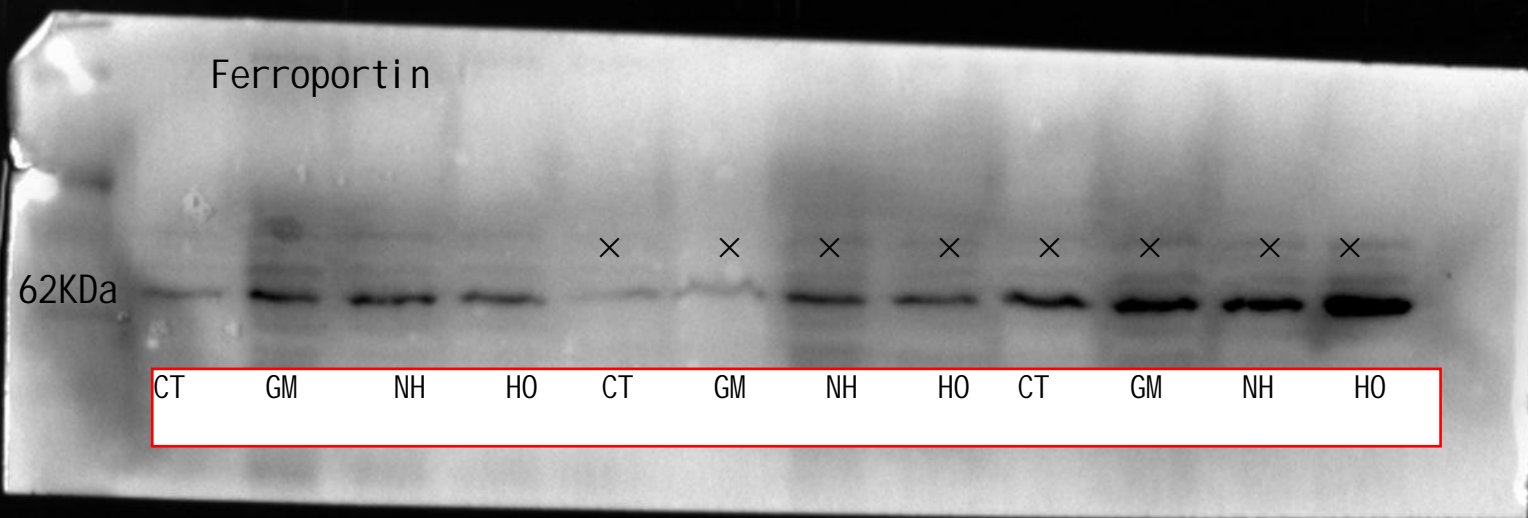

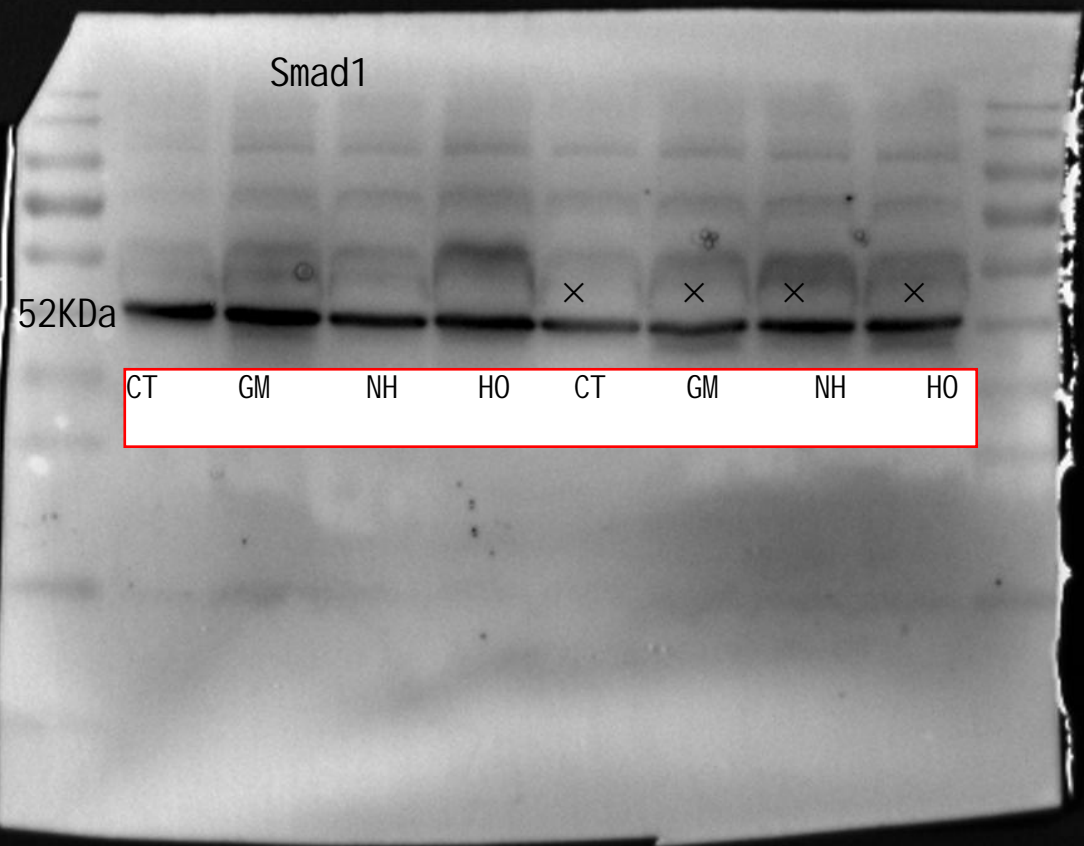

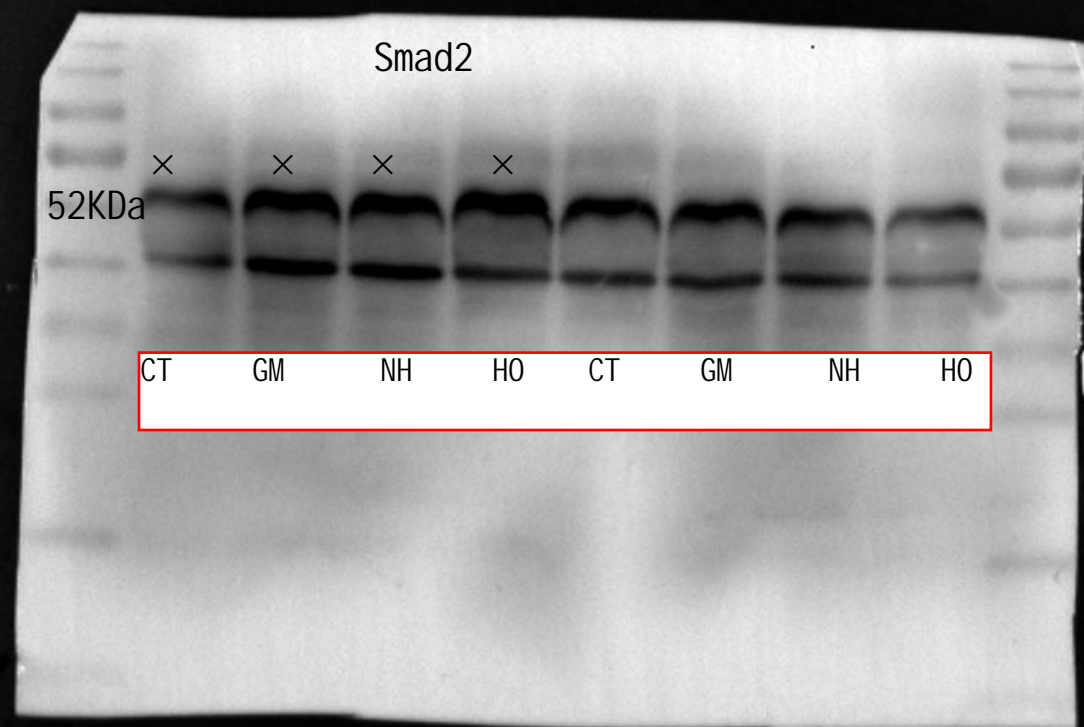

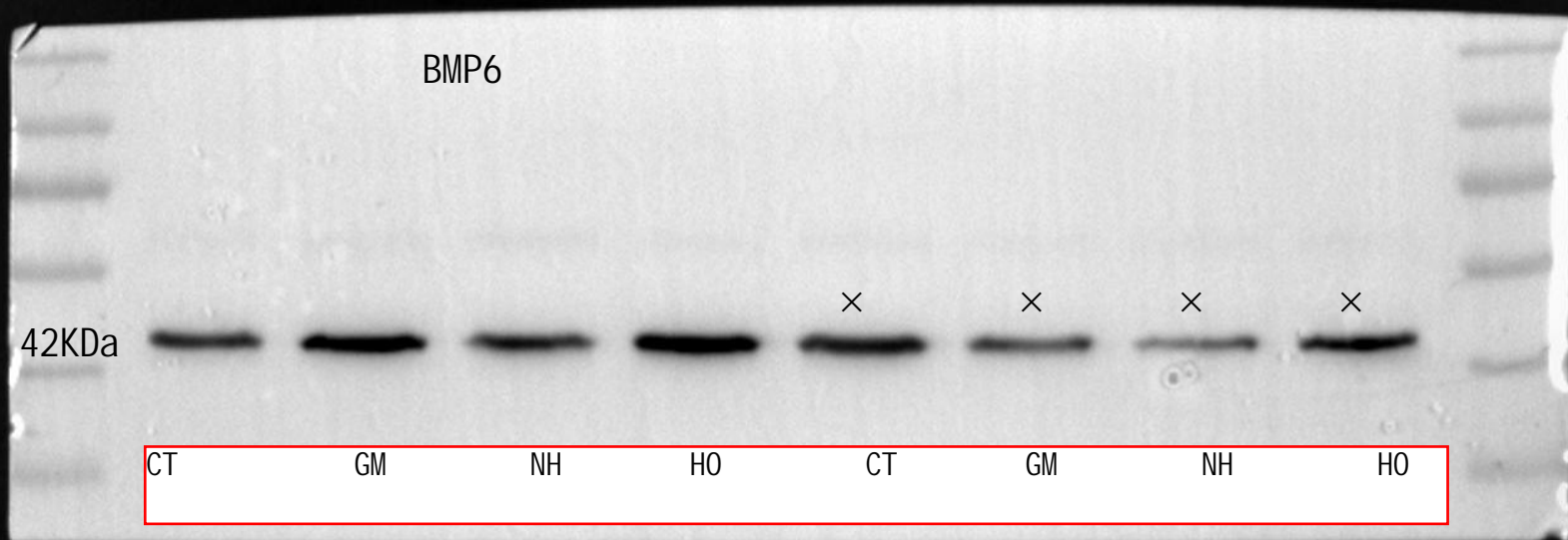

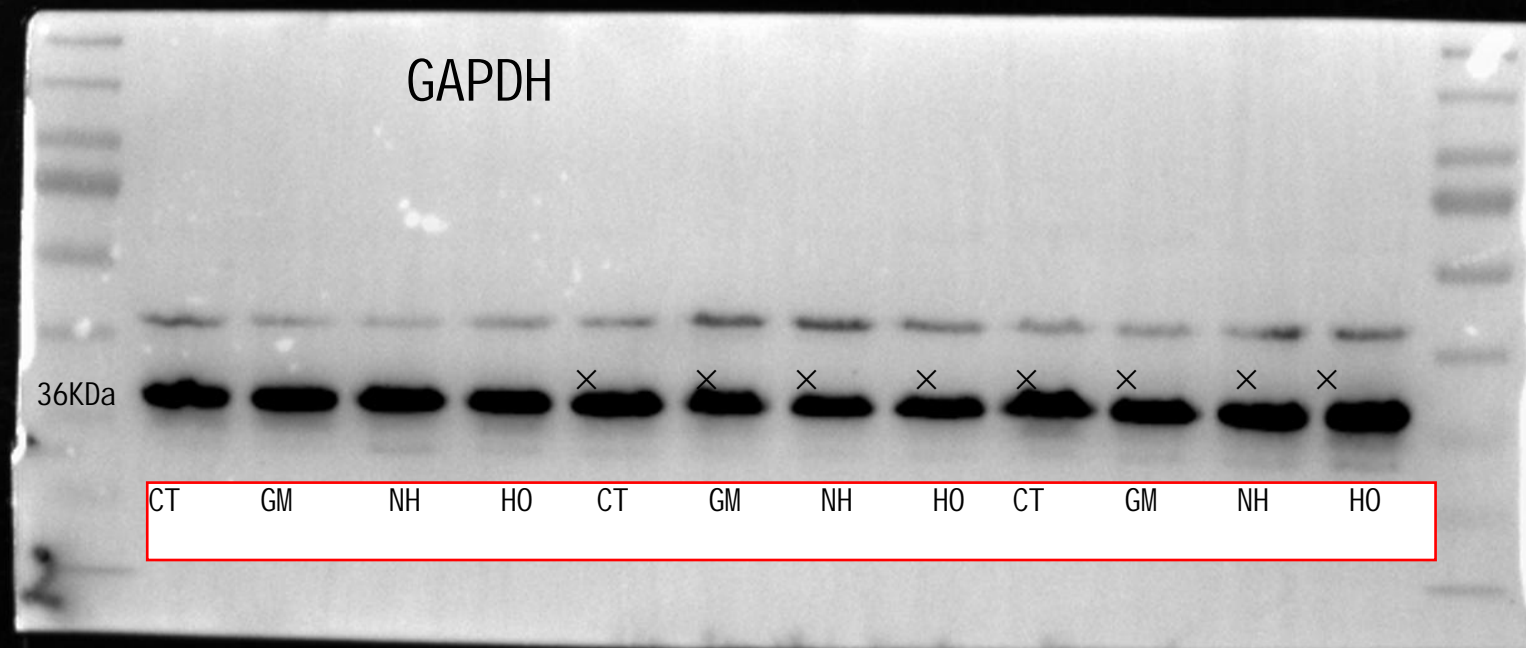

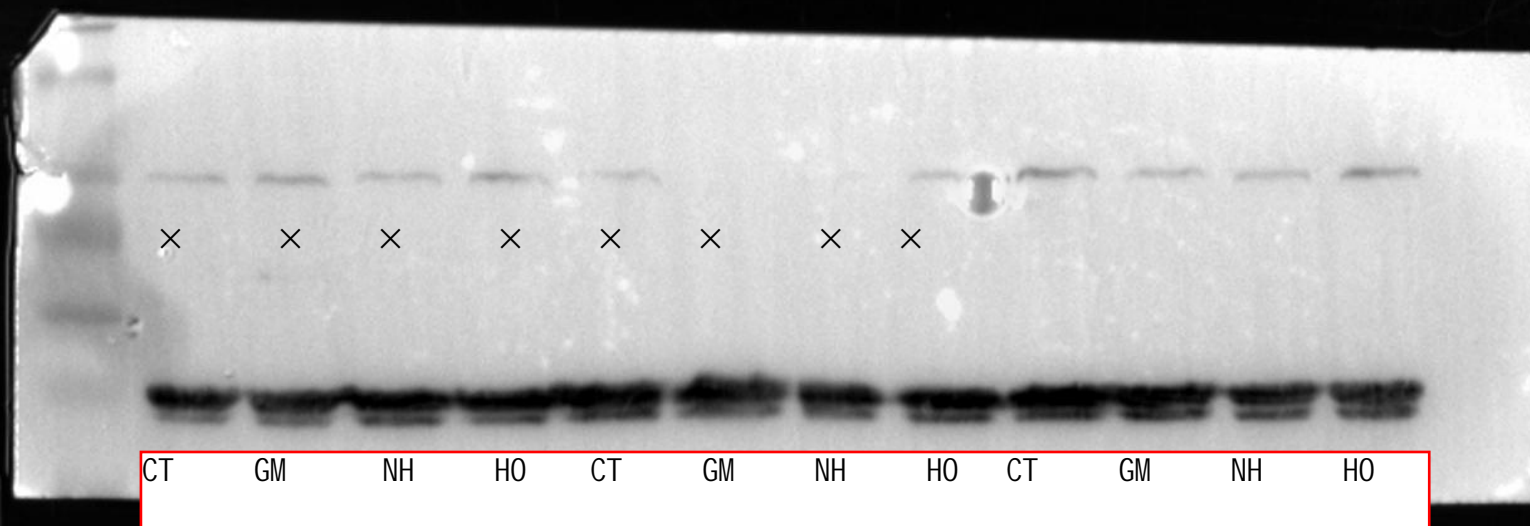

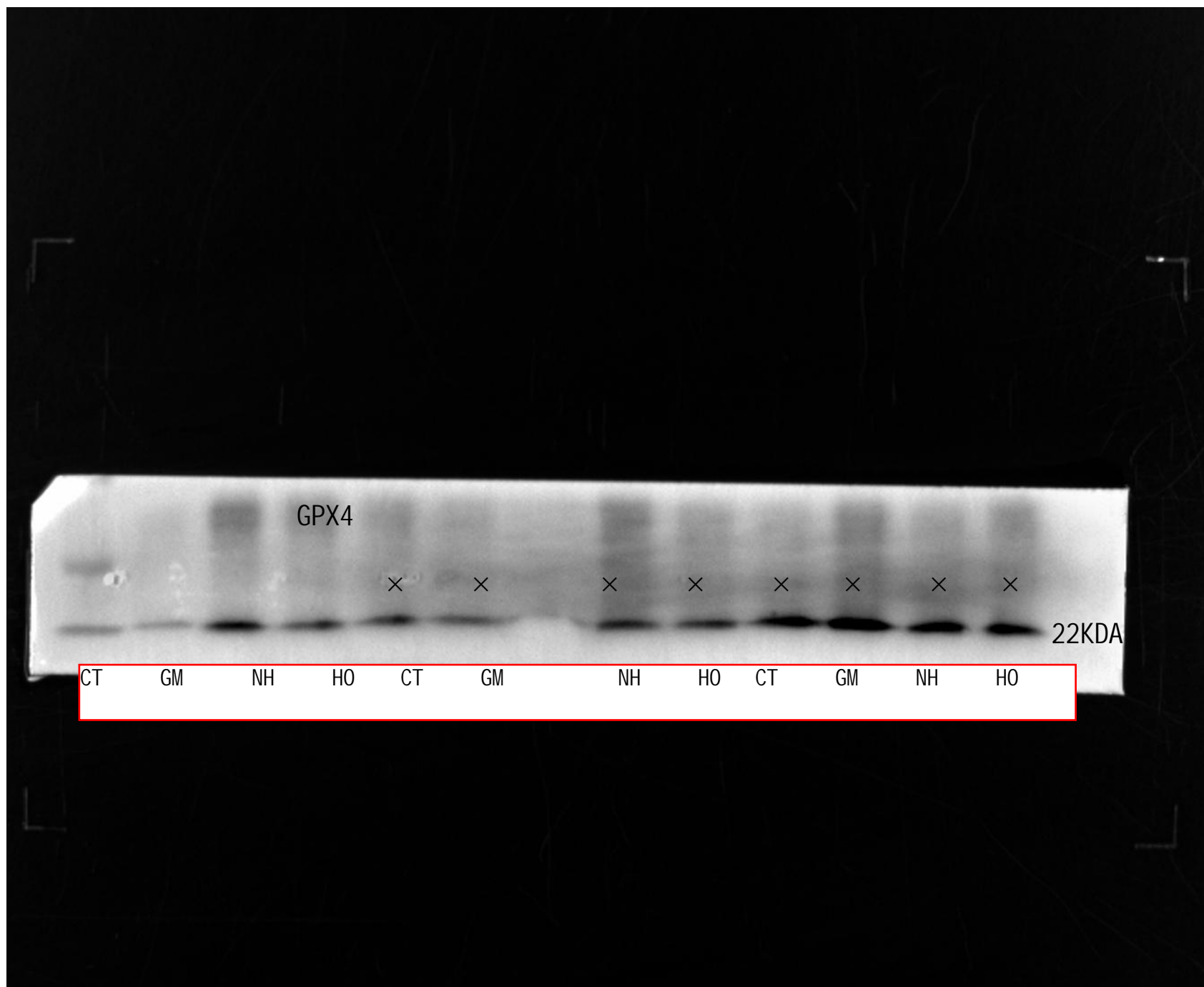

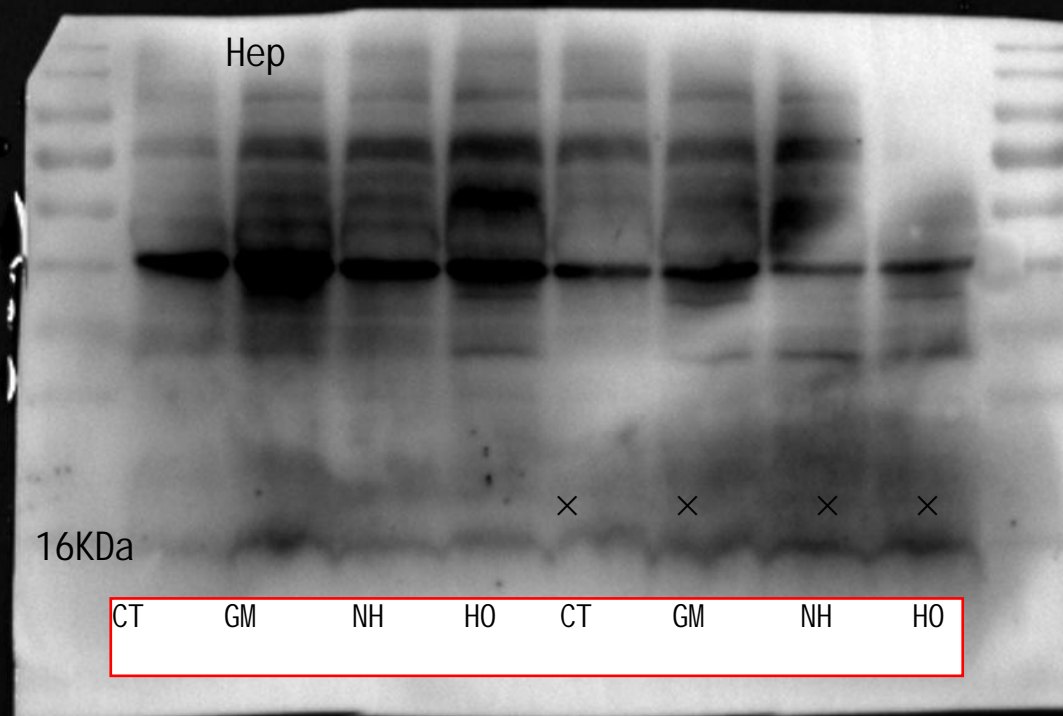

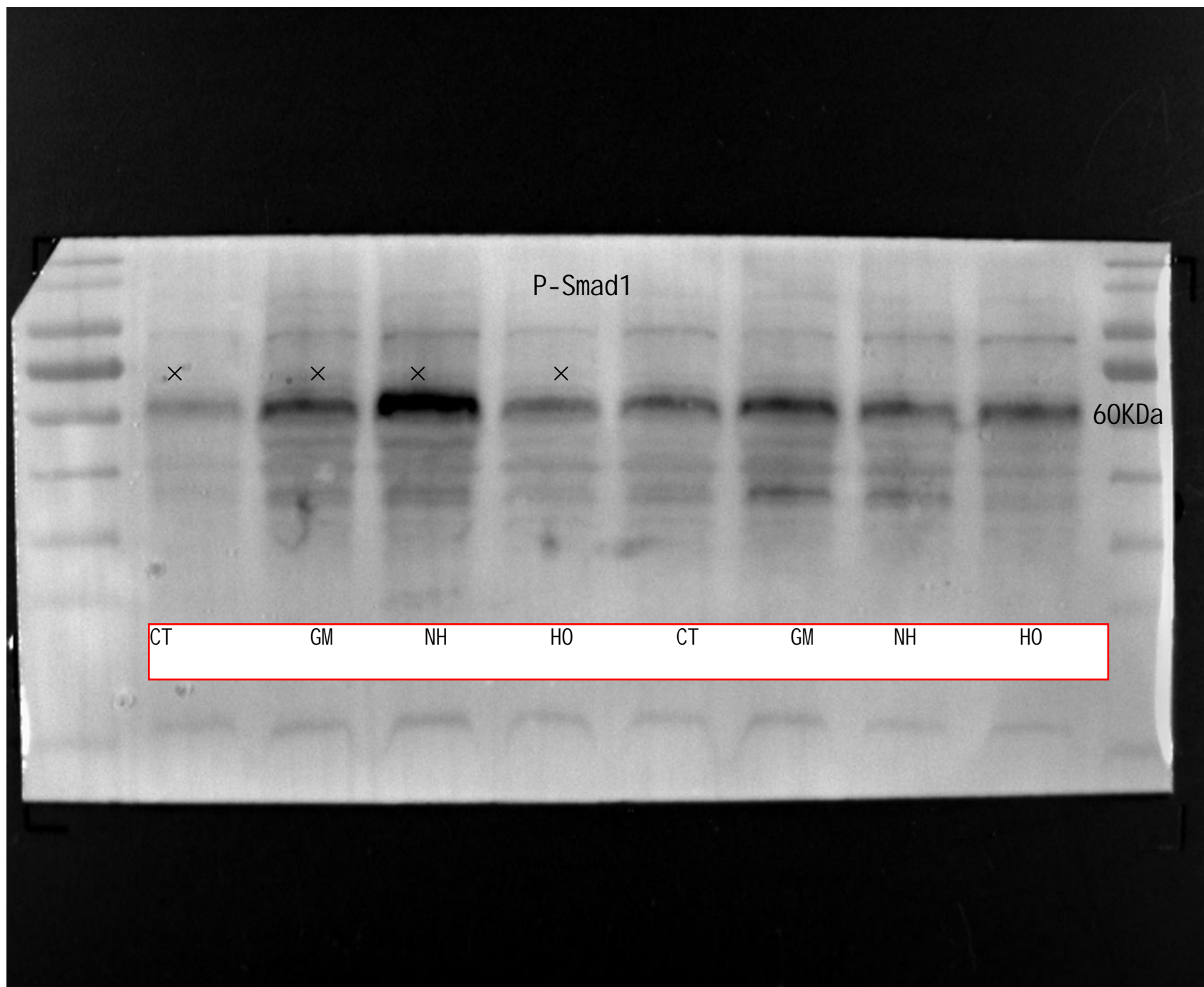

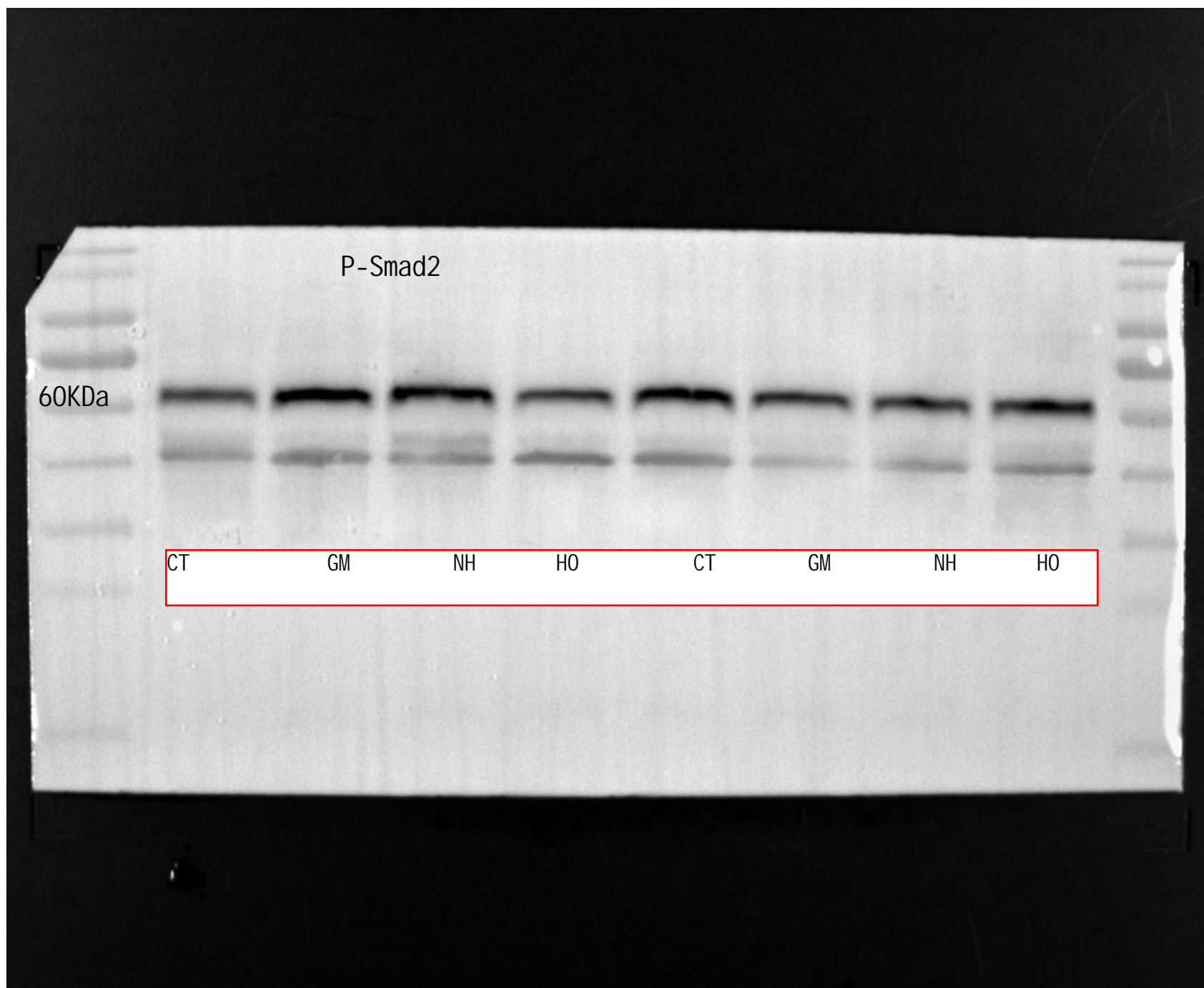

Supplement: S1 Images — (PDF) [file pone.0339455.s003.pdf]

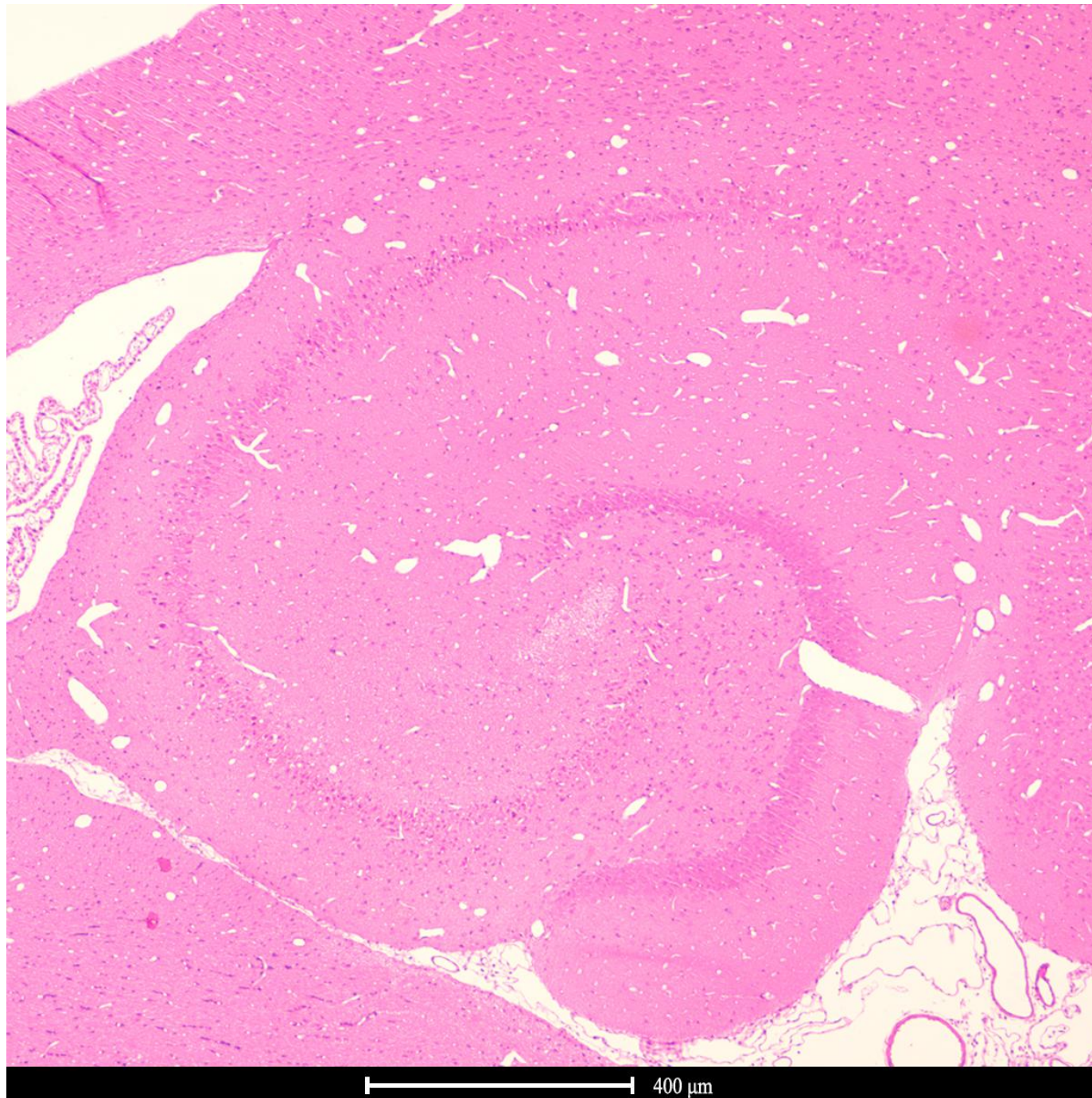

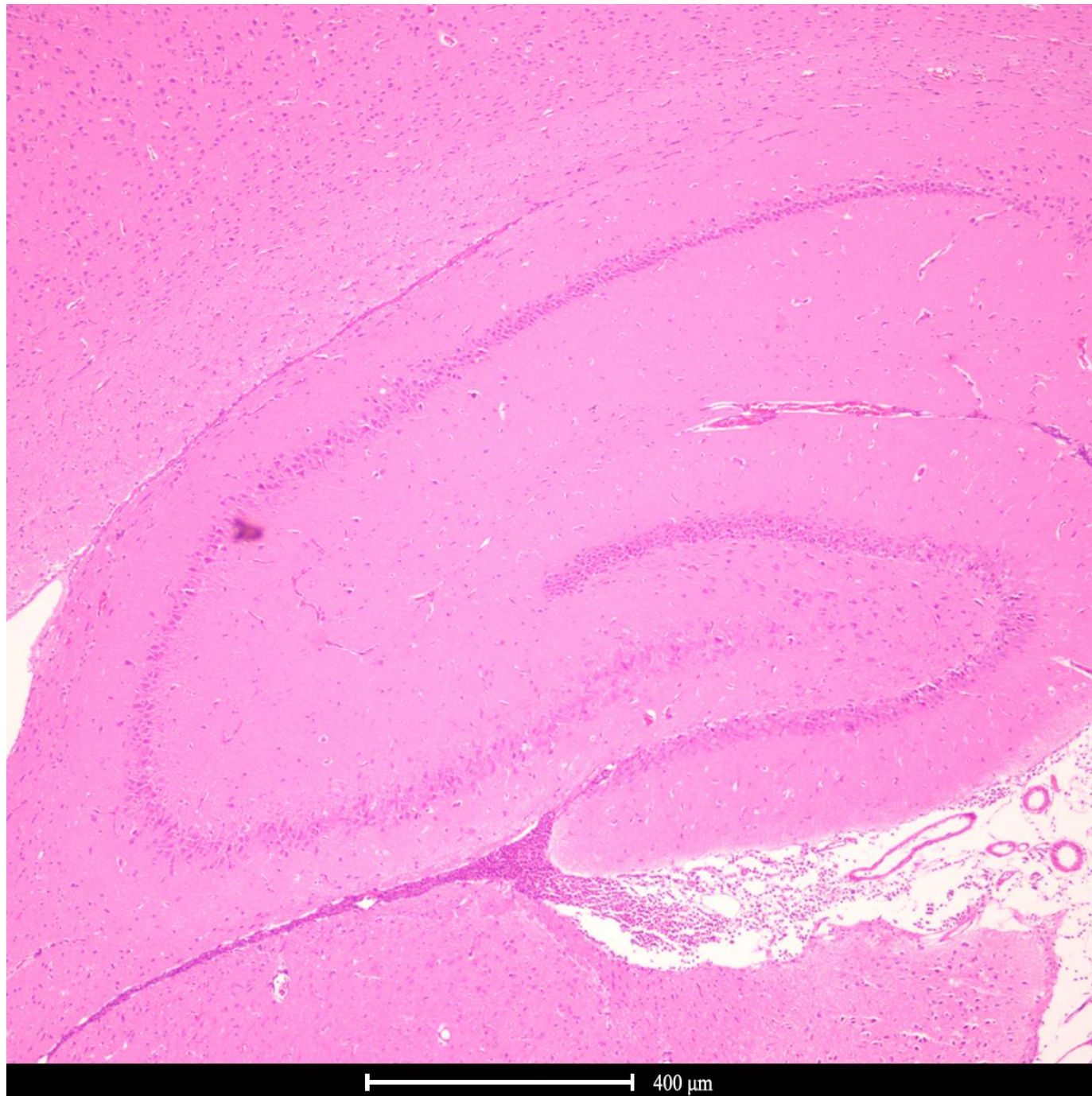

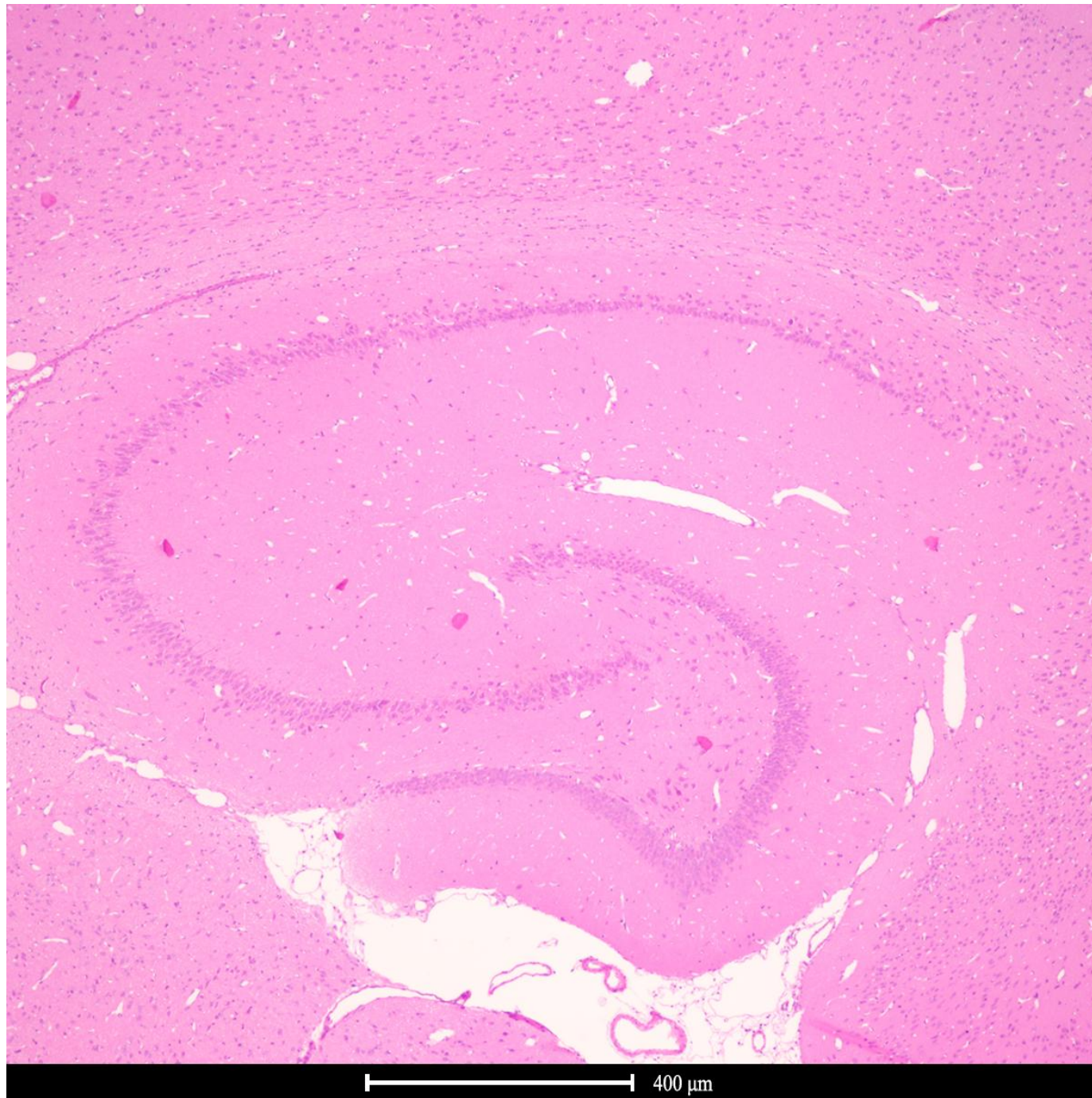

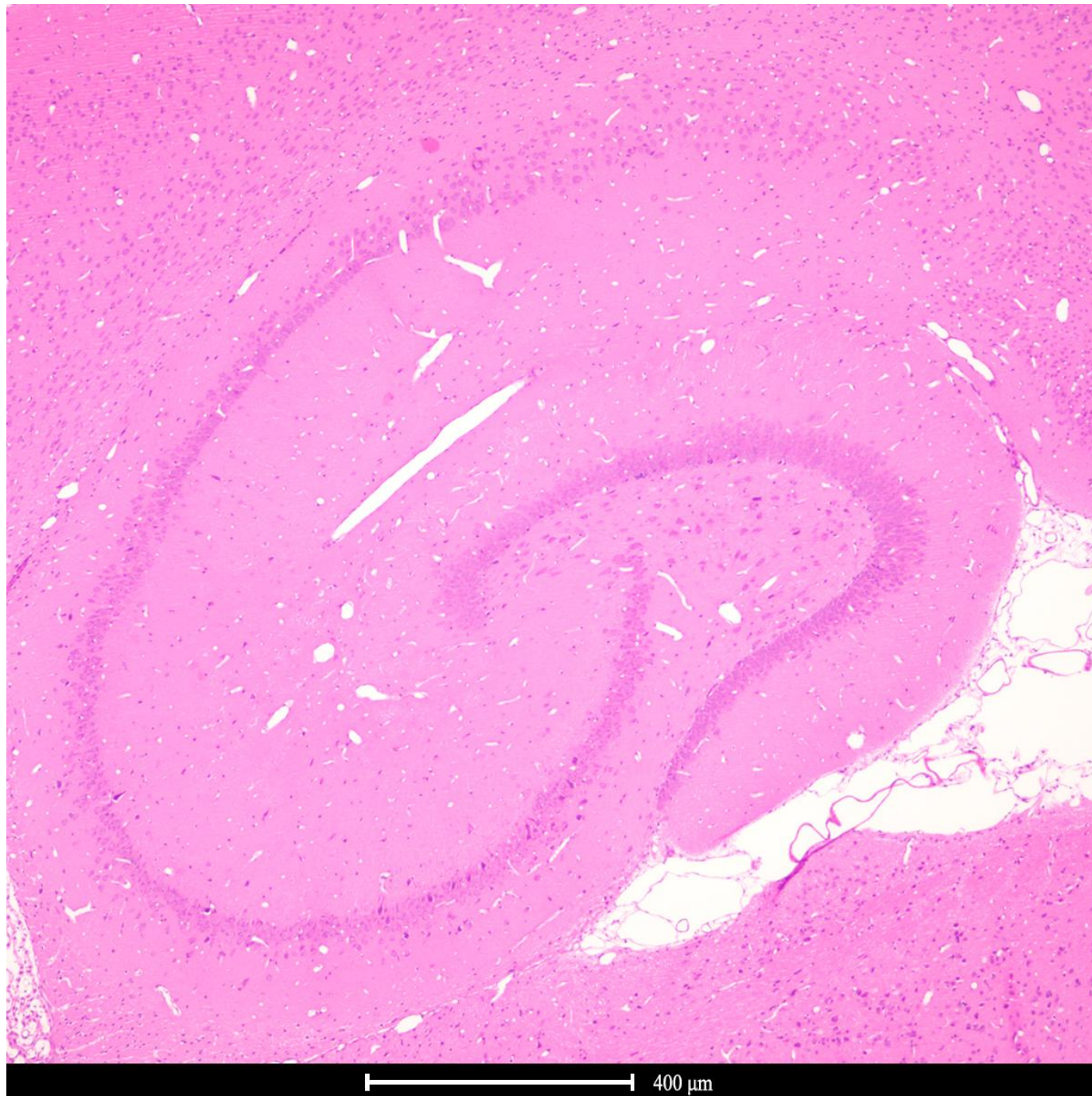

Supplement: S1 Data — (ZIP) [file pone.0339455.s004.zip › HE.pdf]

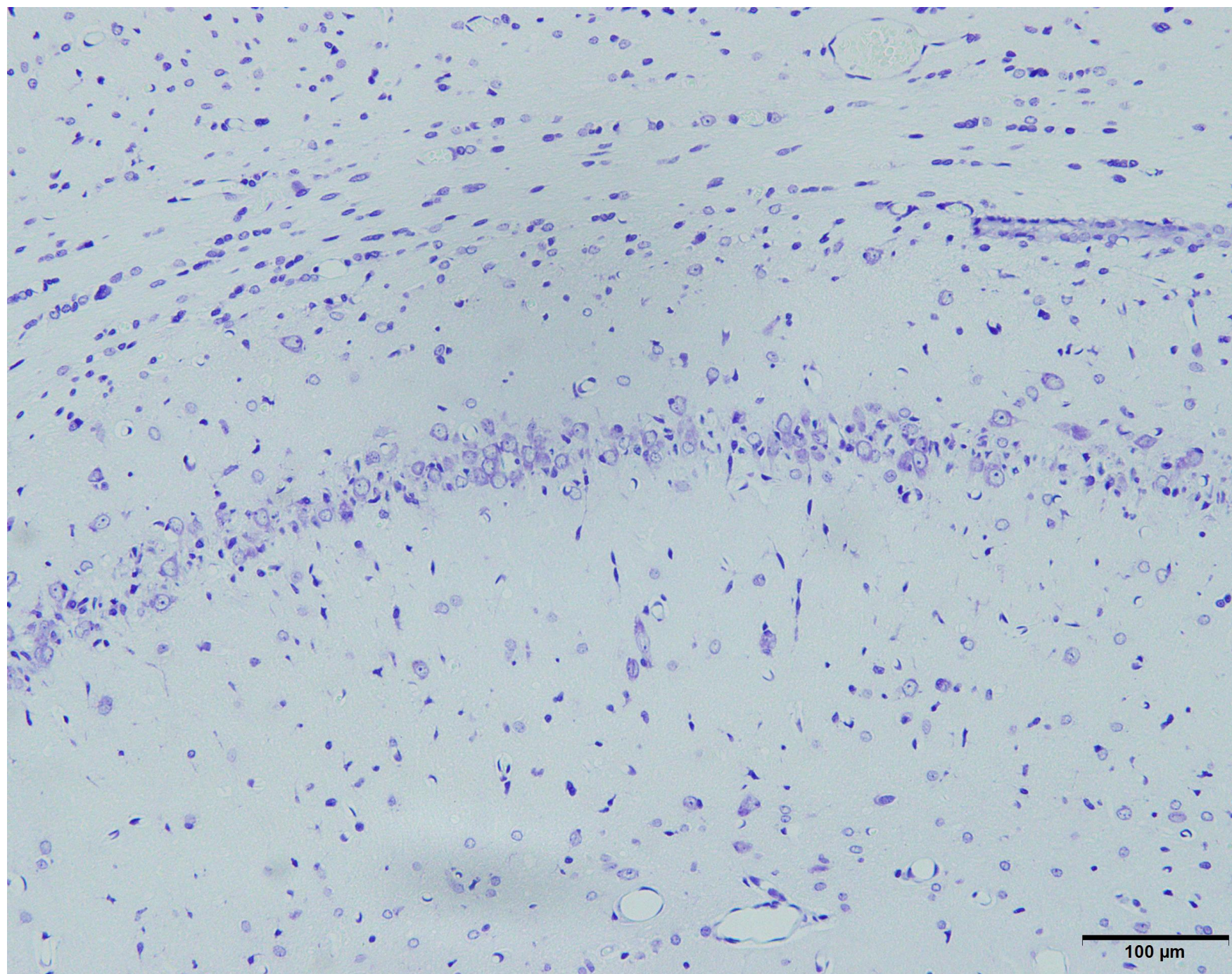

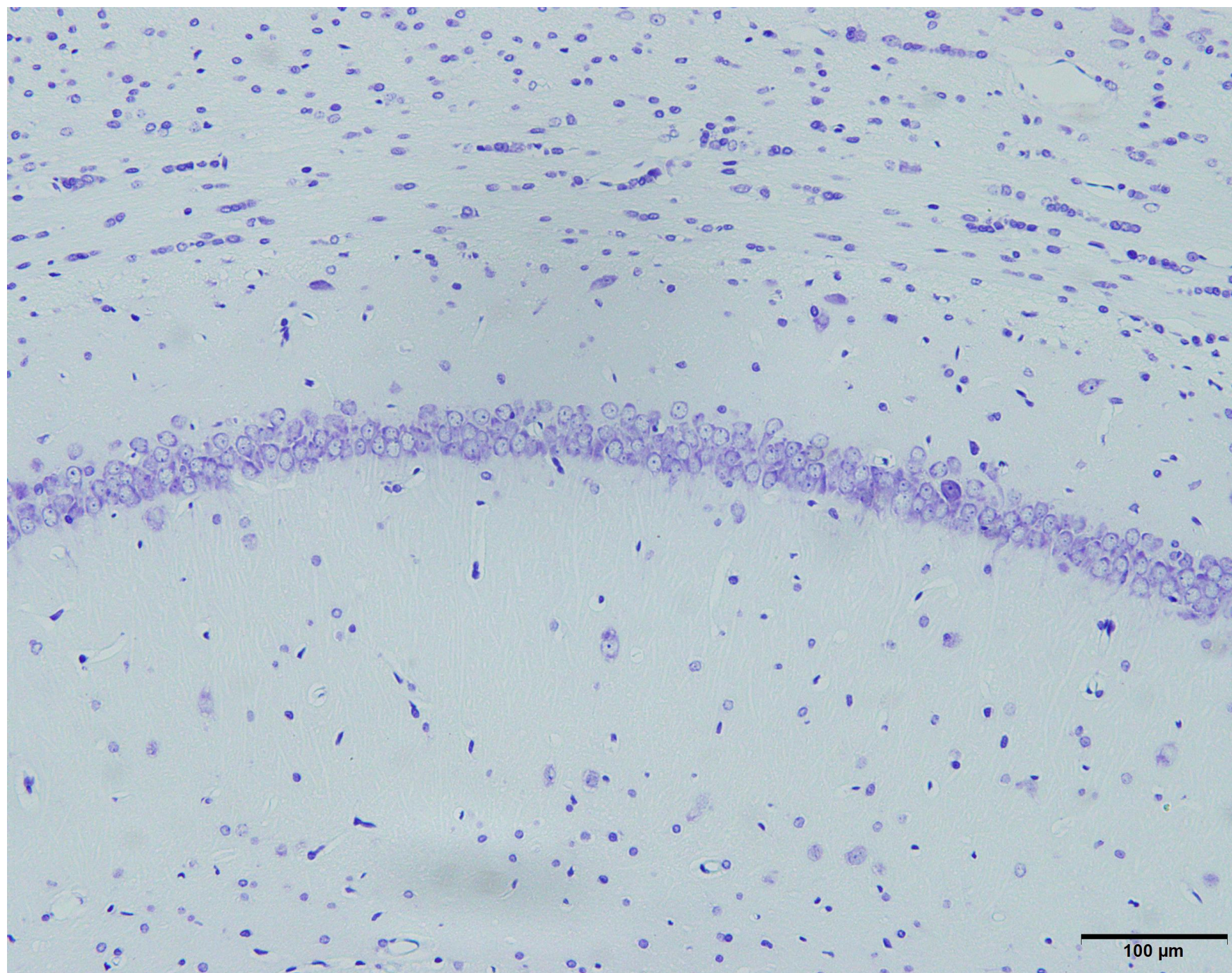

100  $\mu$ m

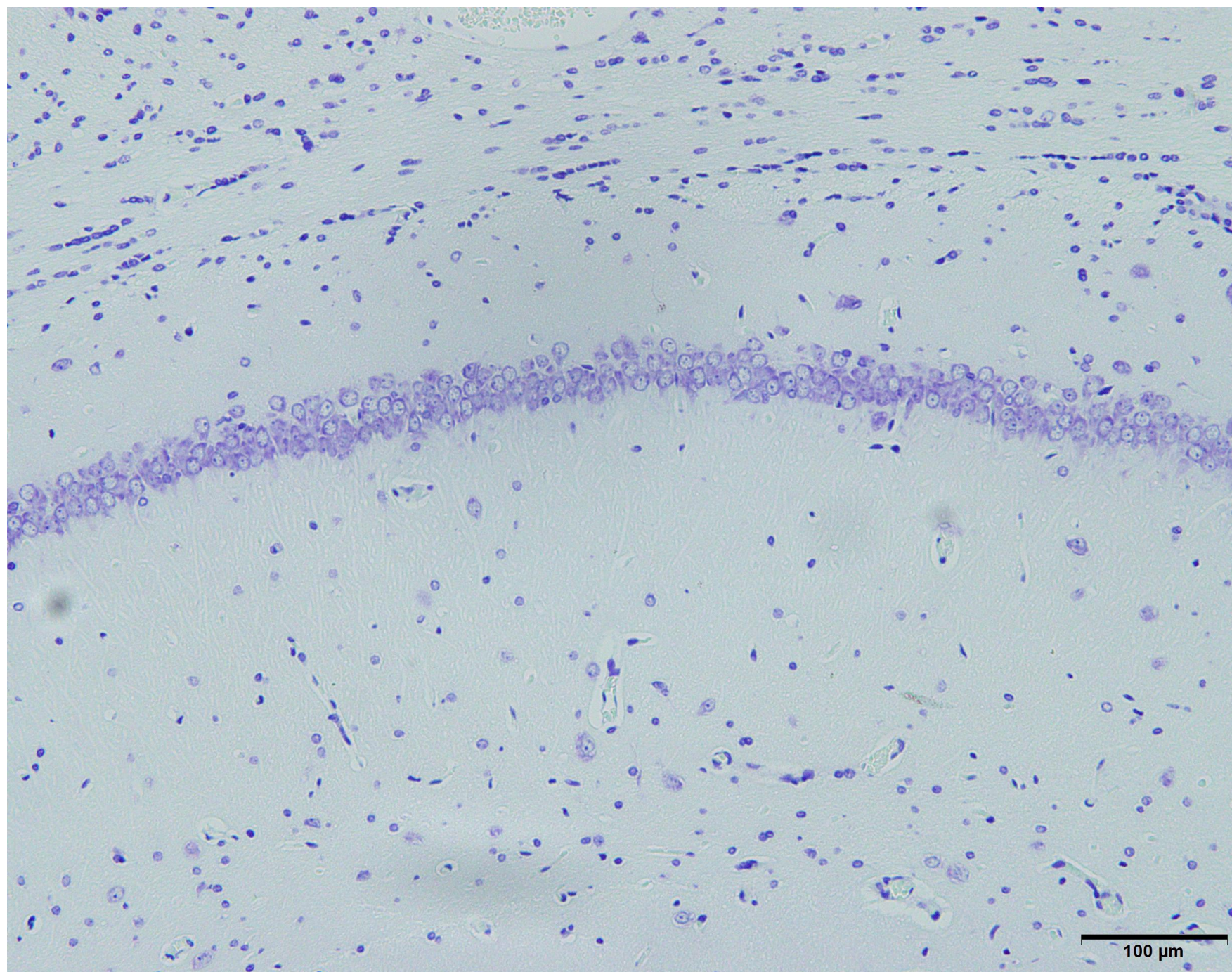

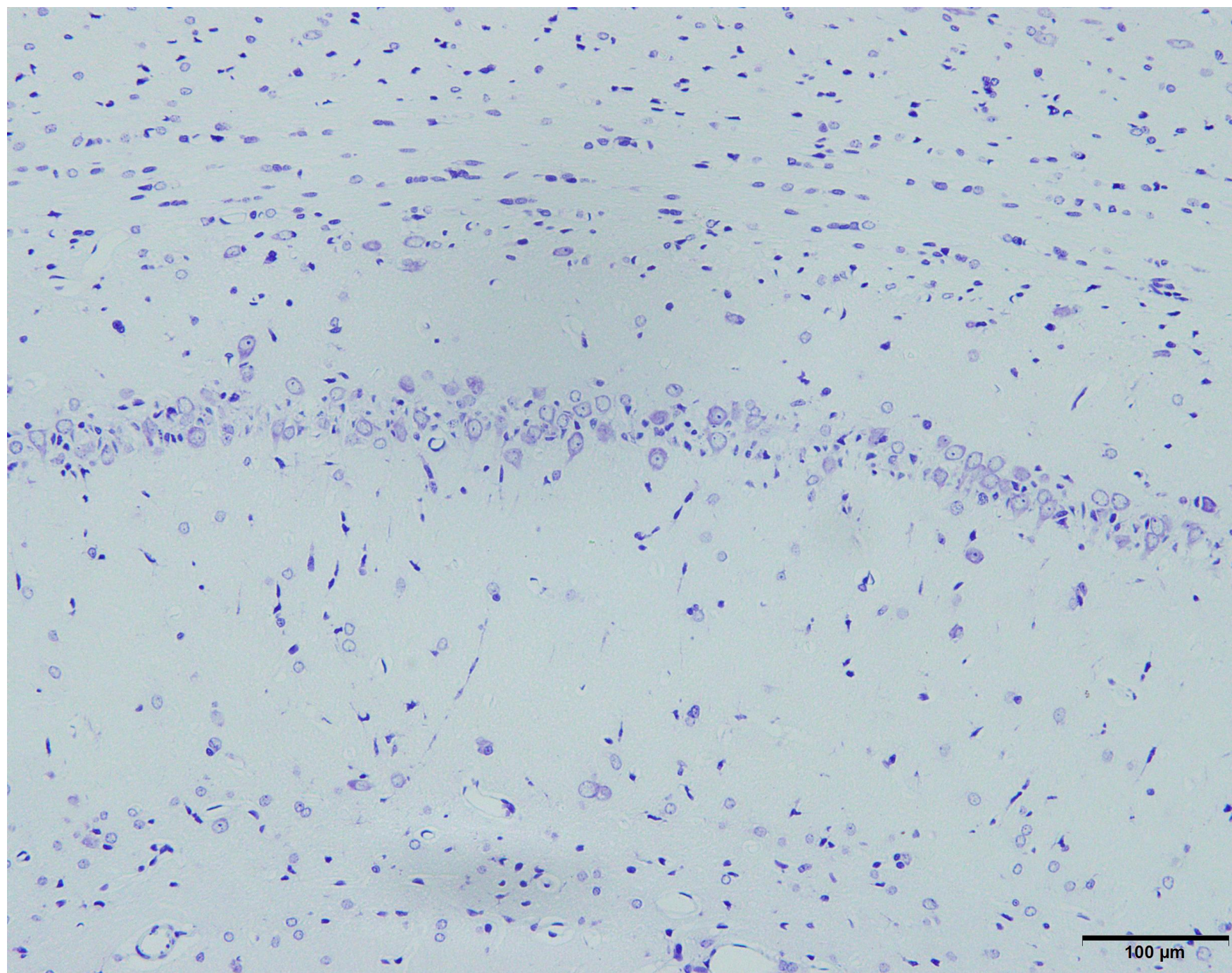

Supplement: S1 Data — (ZIP) [file pone.0339455.s004.zip › Nissl.pdf]

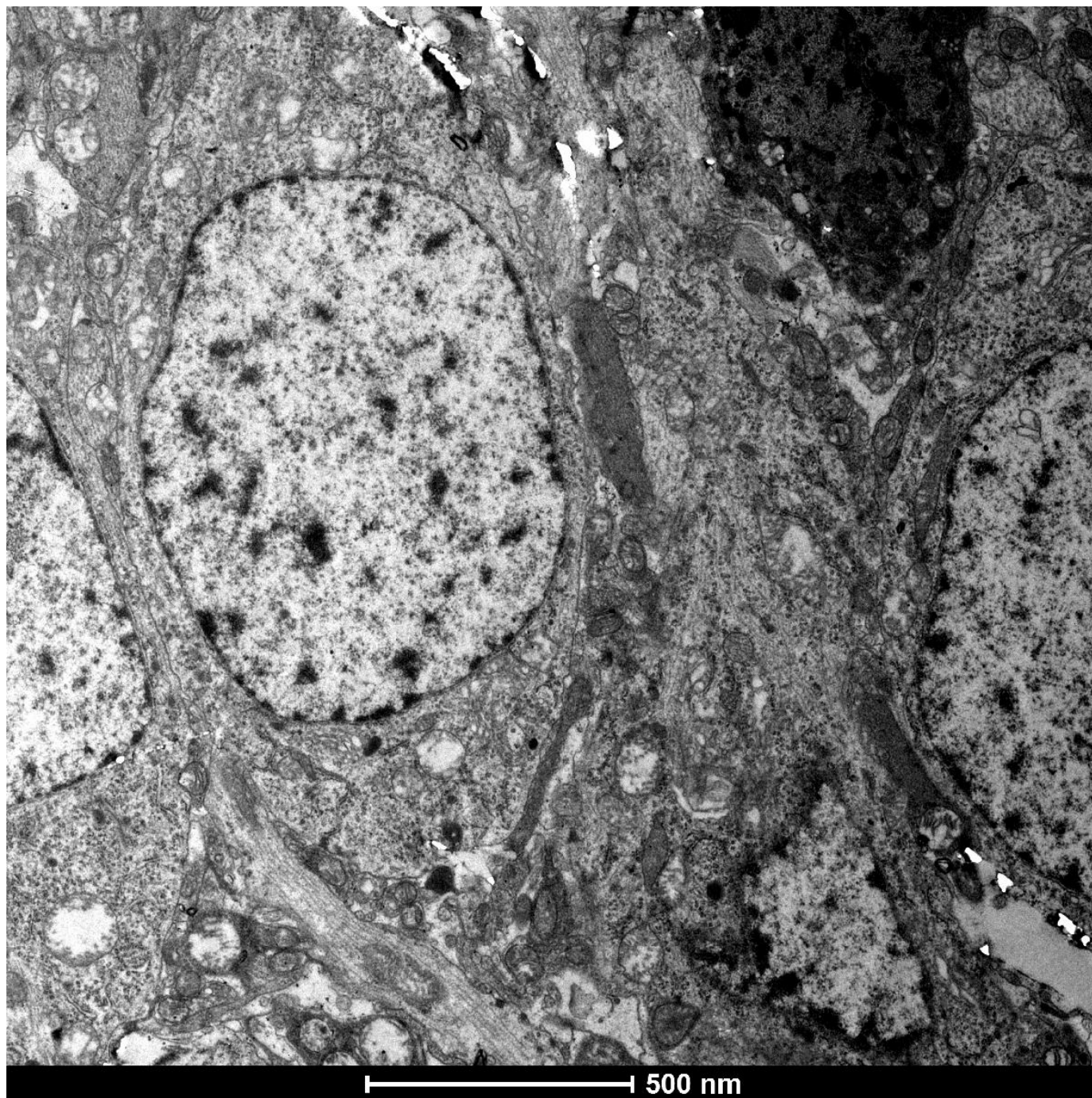

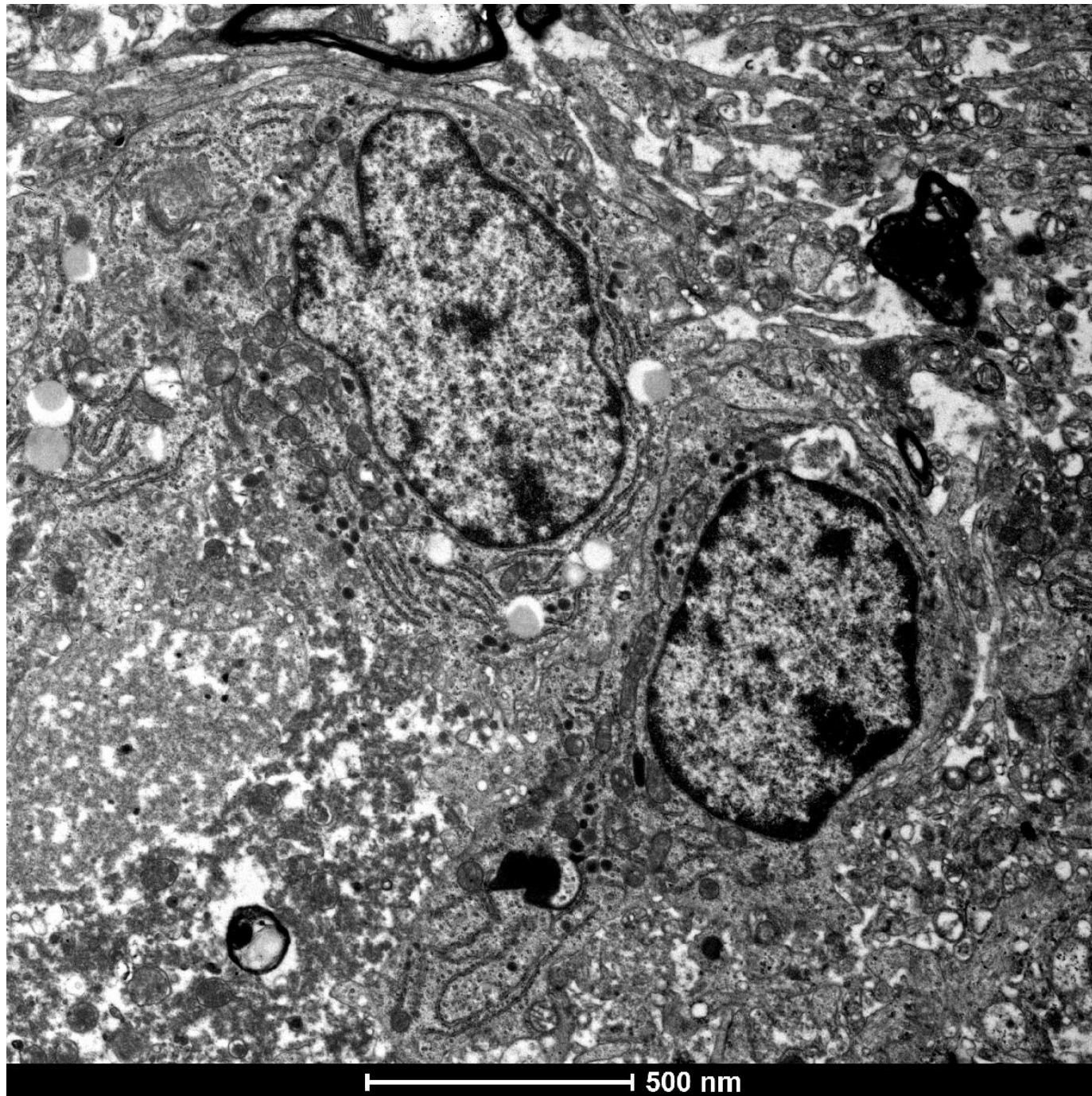

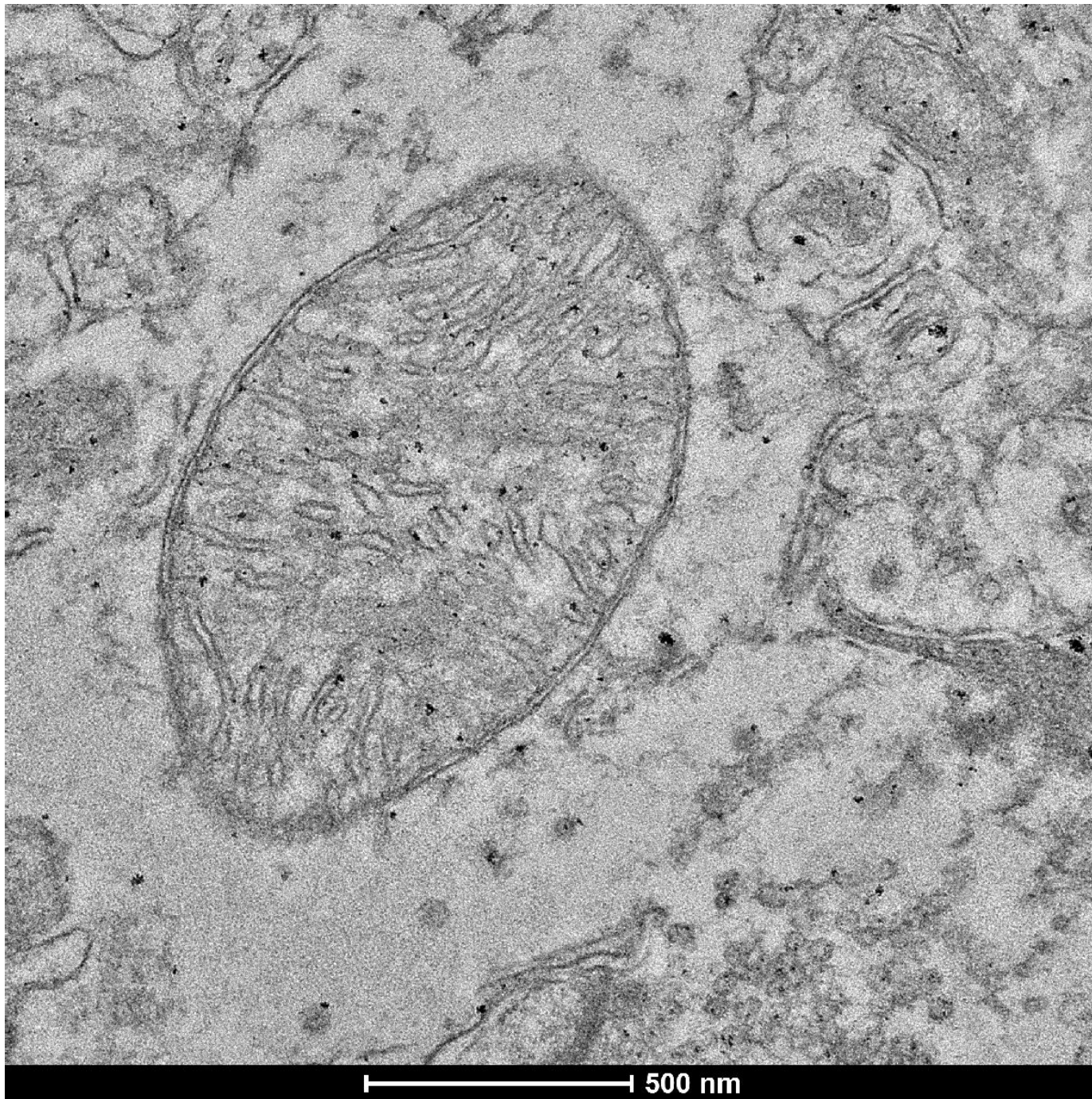

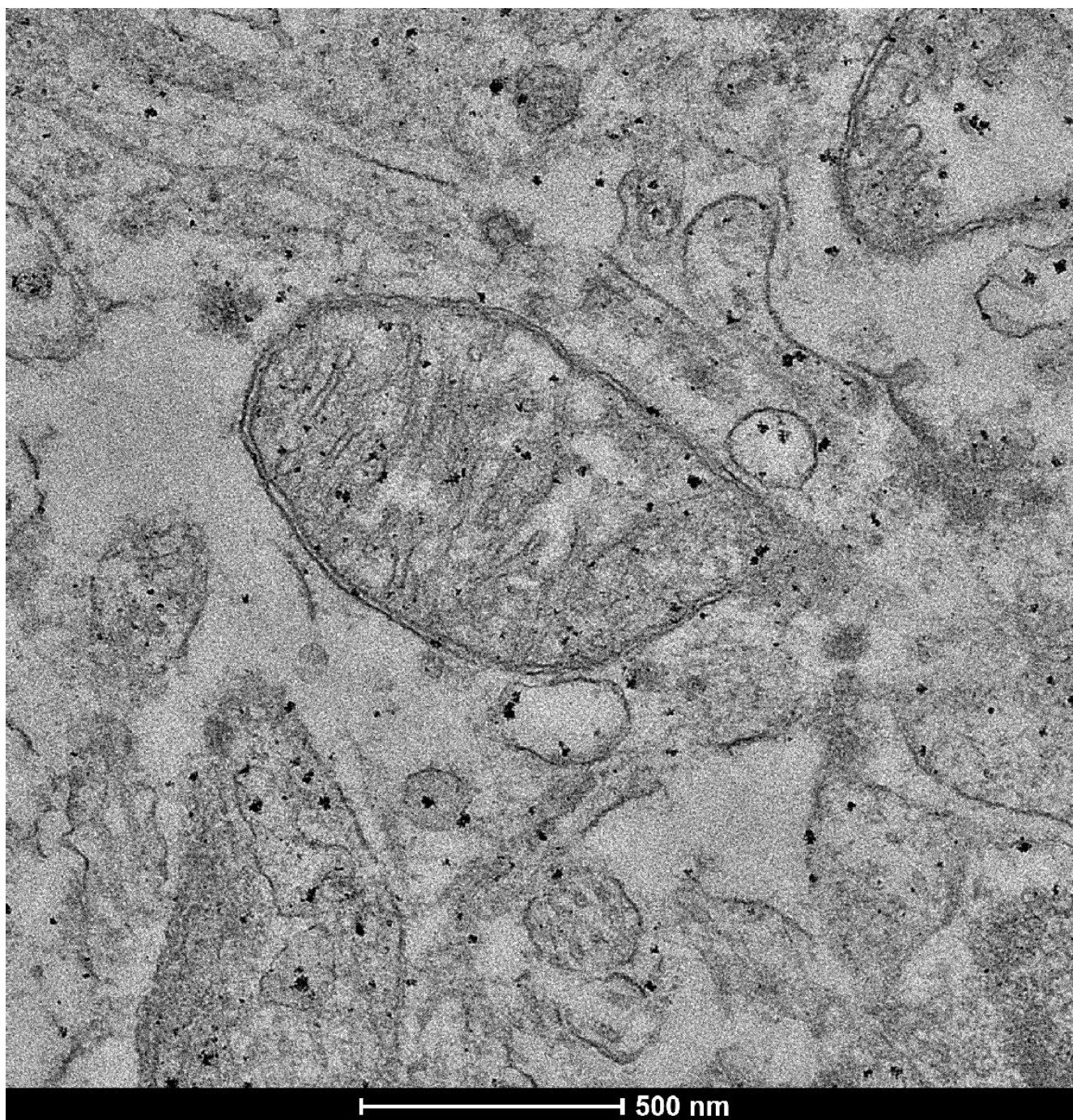

Supplement: S1 Data — (ZIP) [file pone.0339455.s004.zip › TEM.pdf]

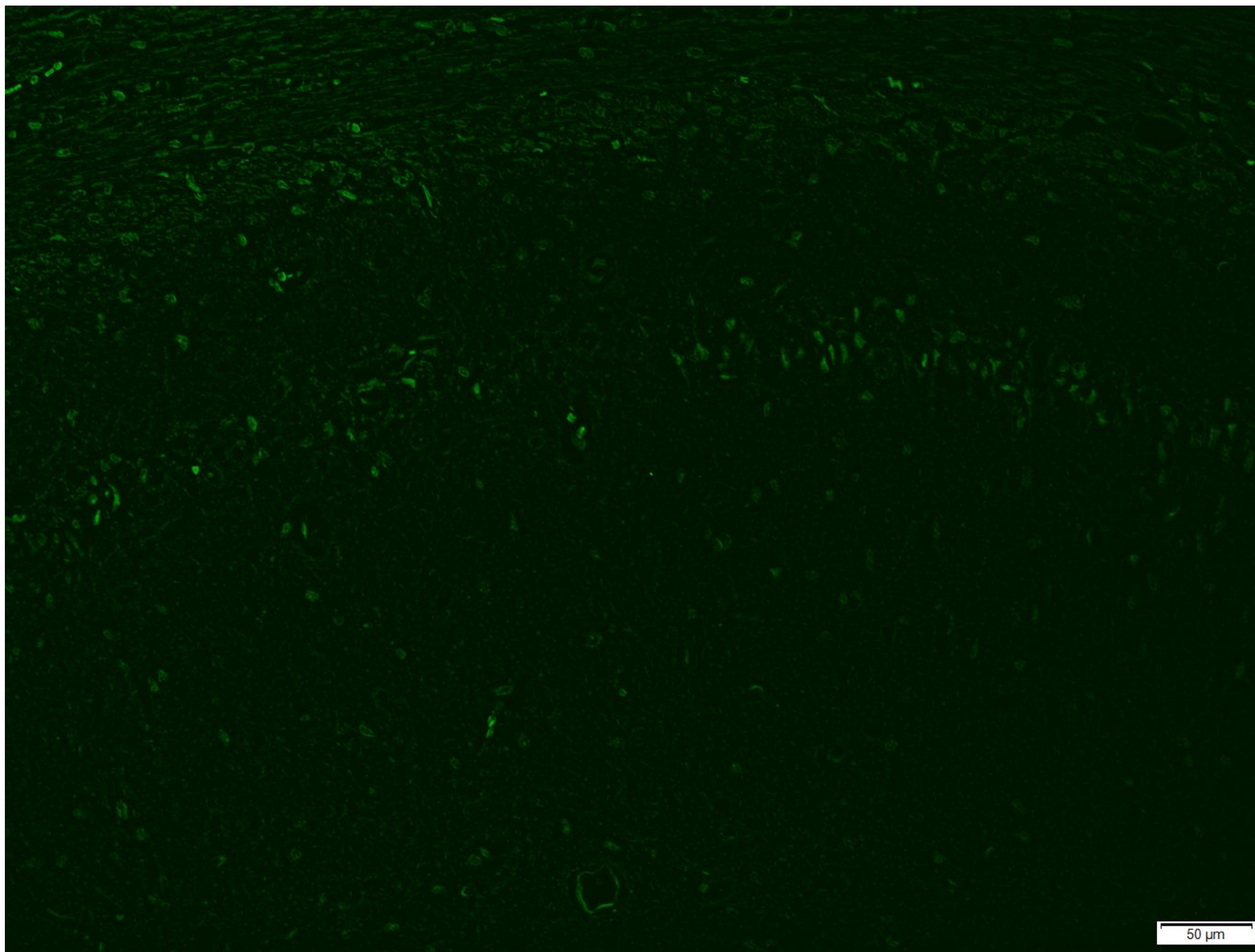

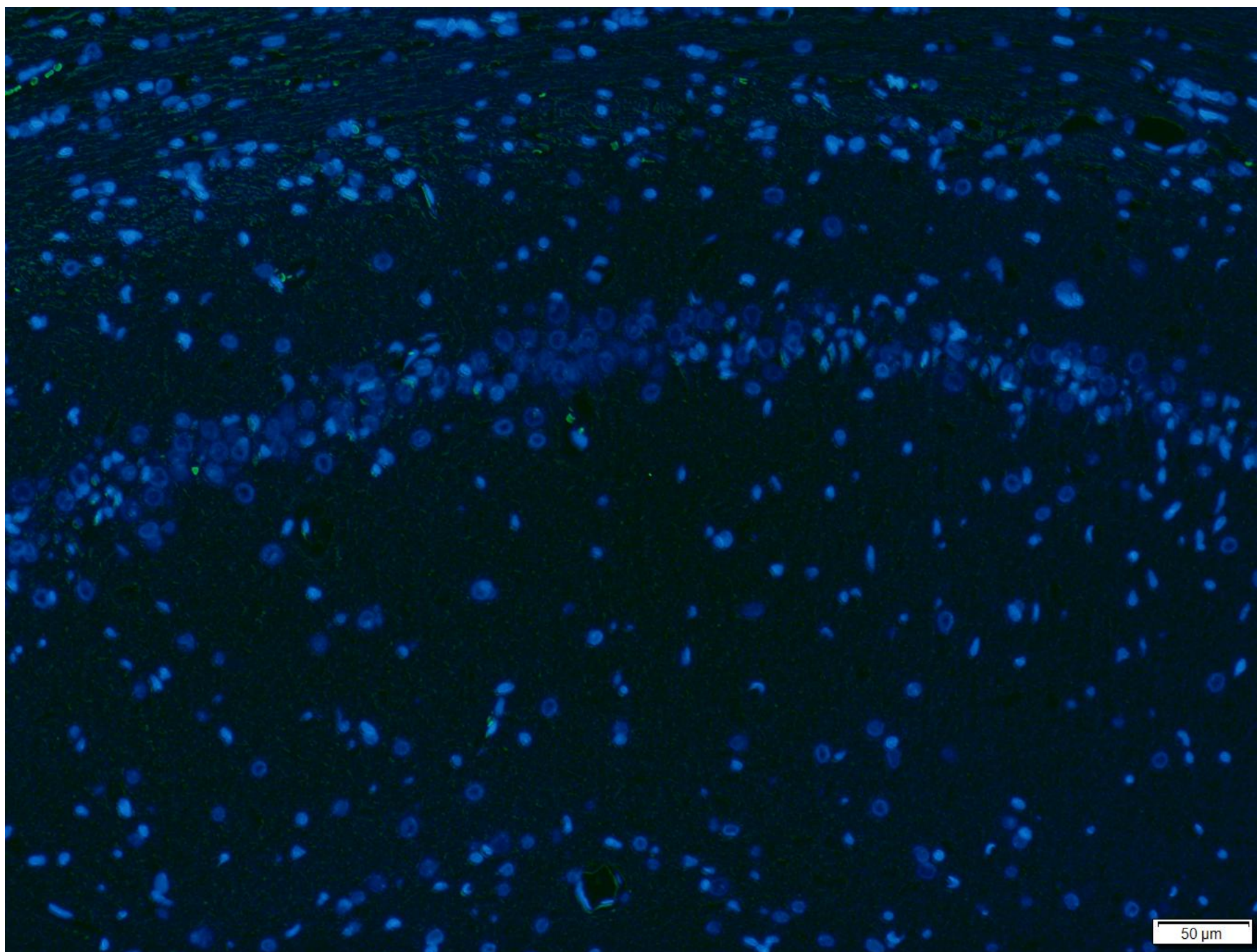

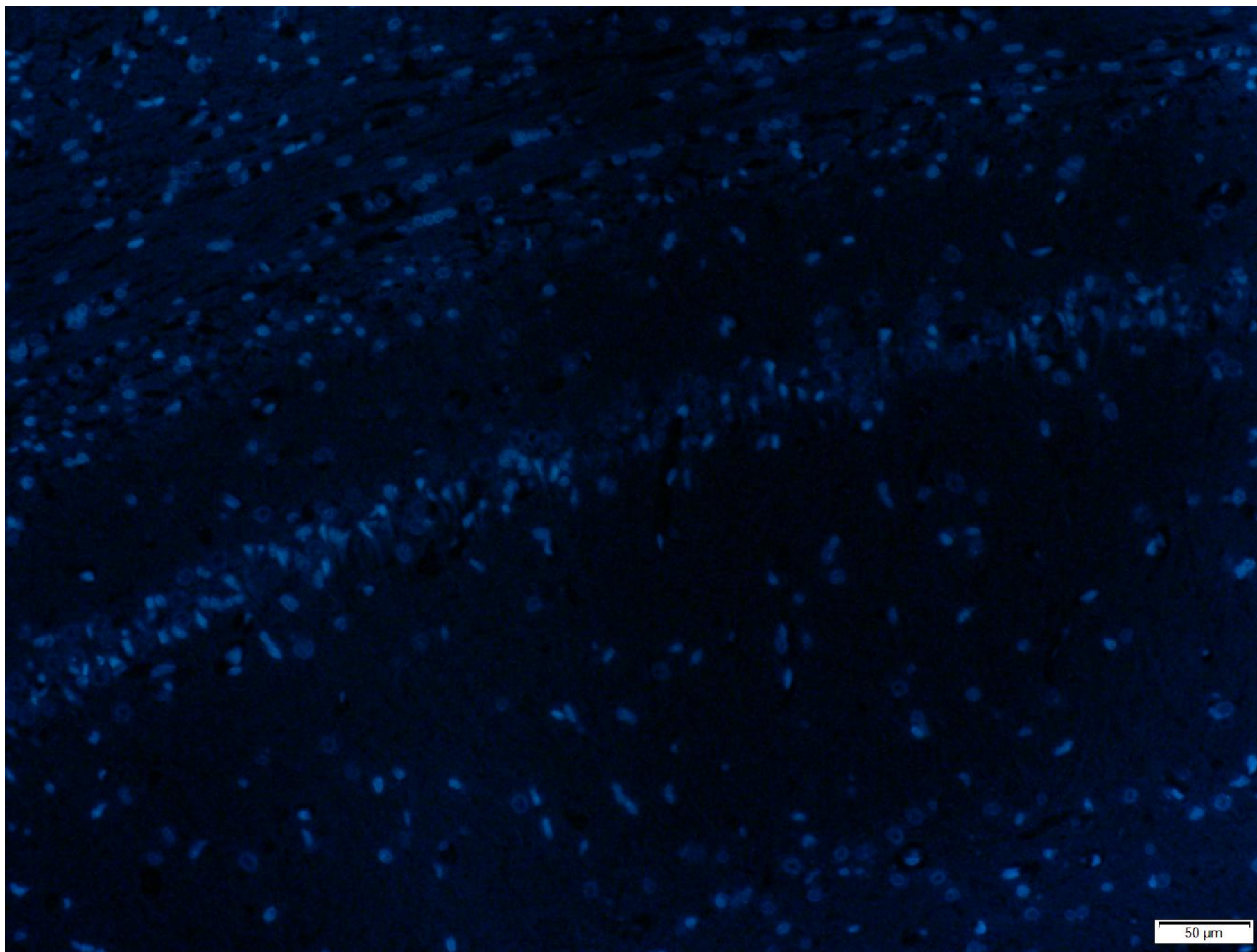

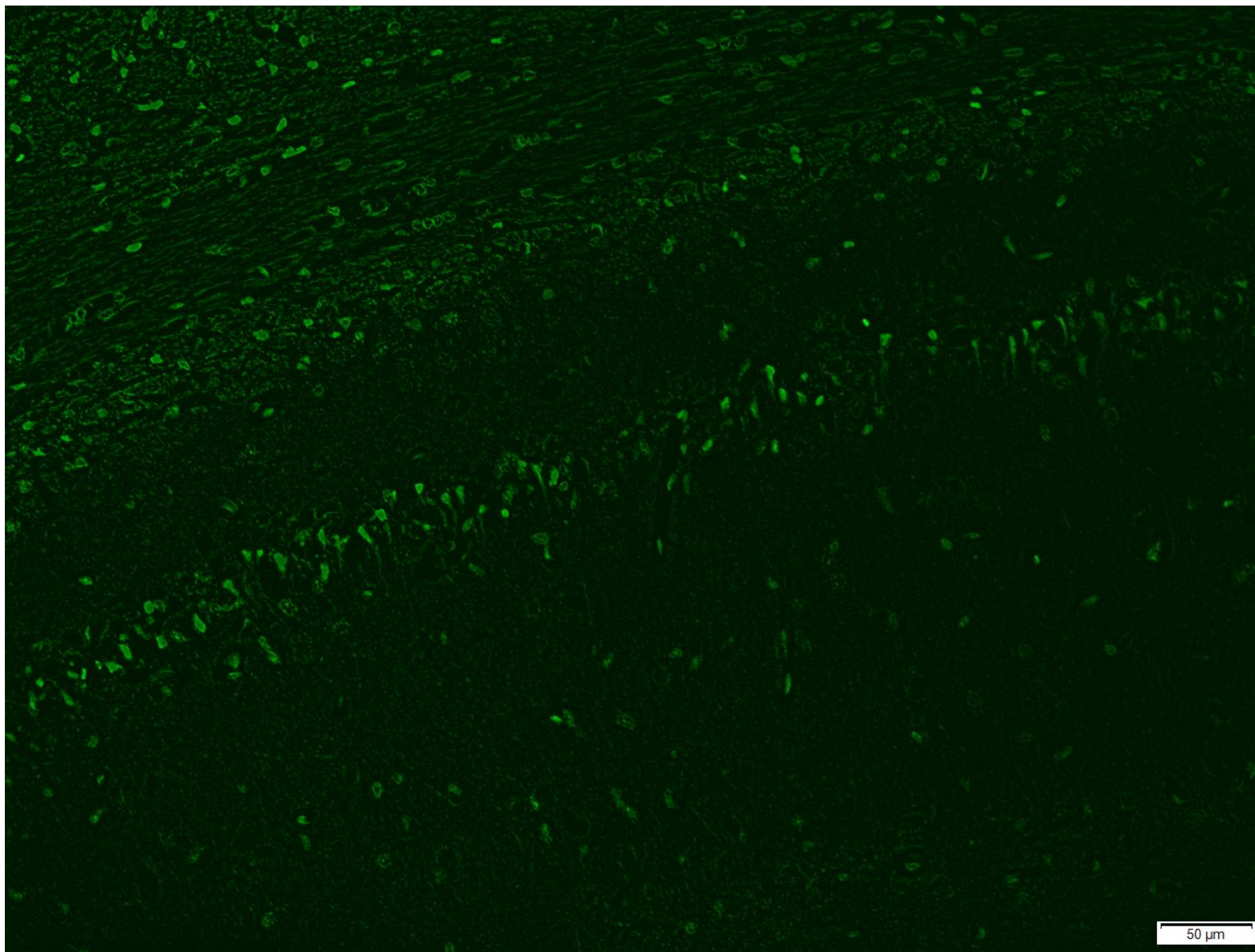

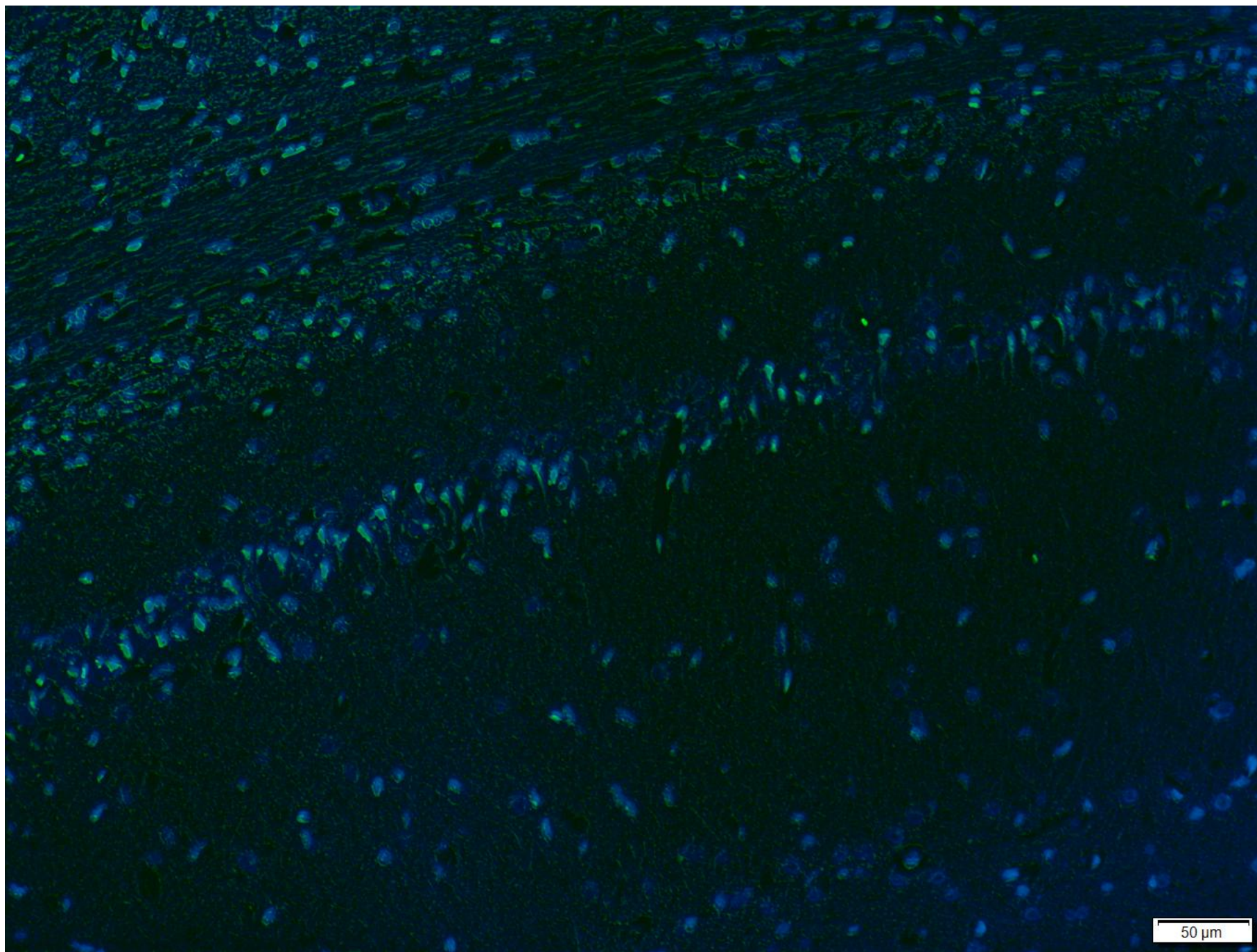

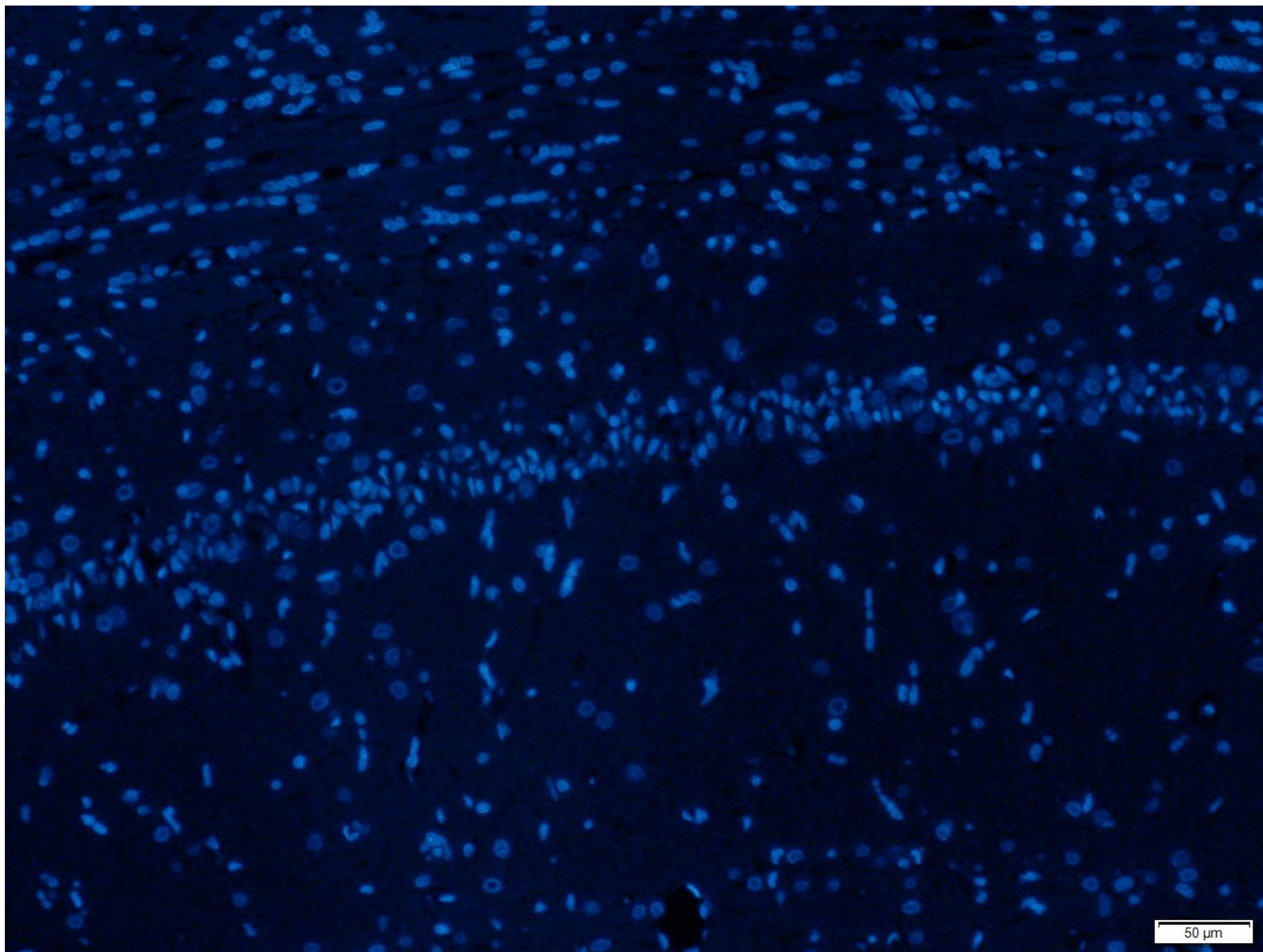

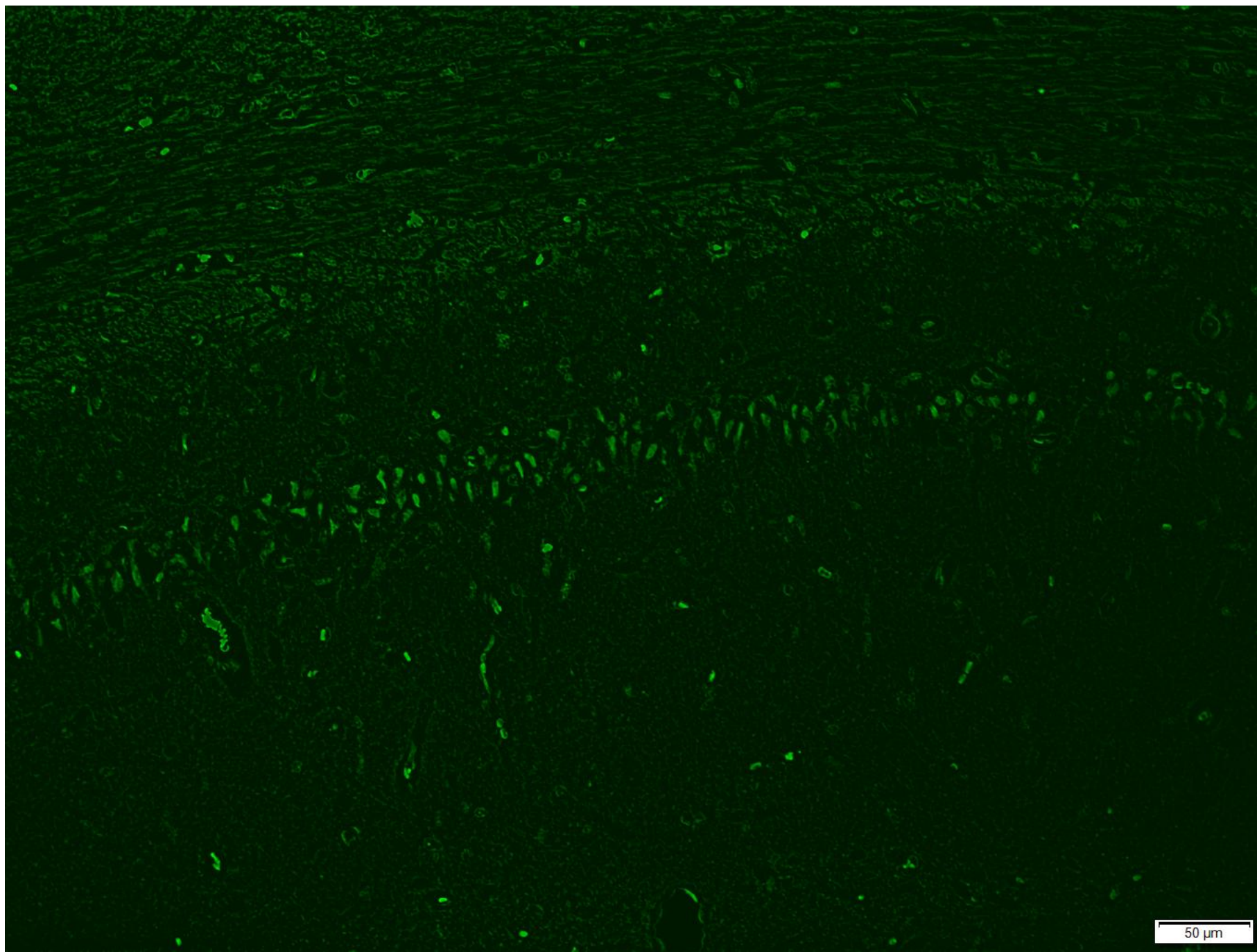

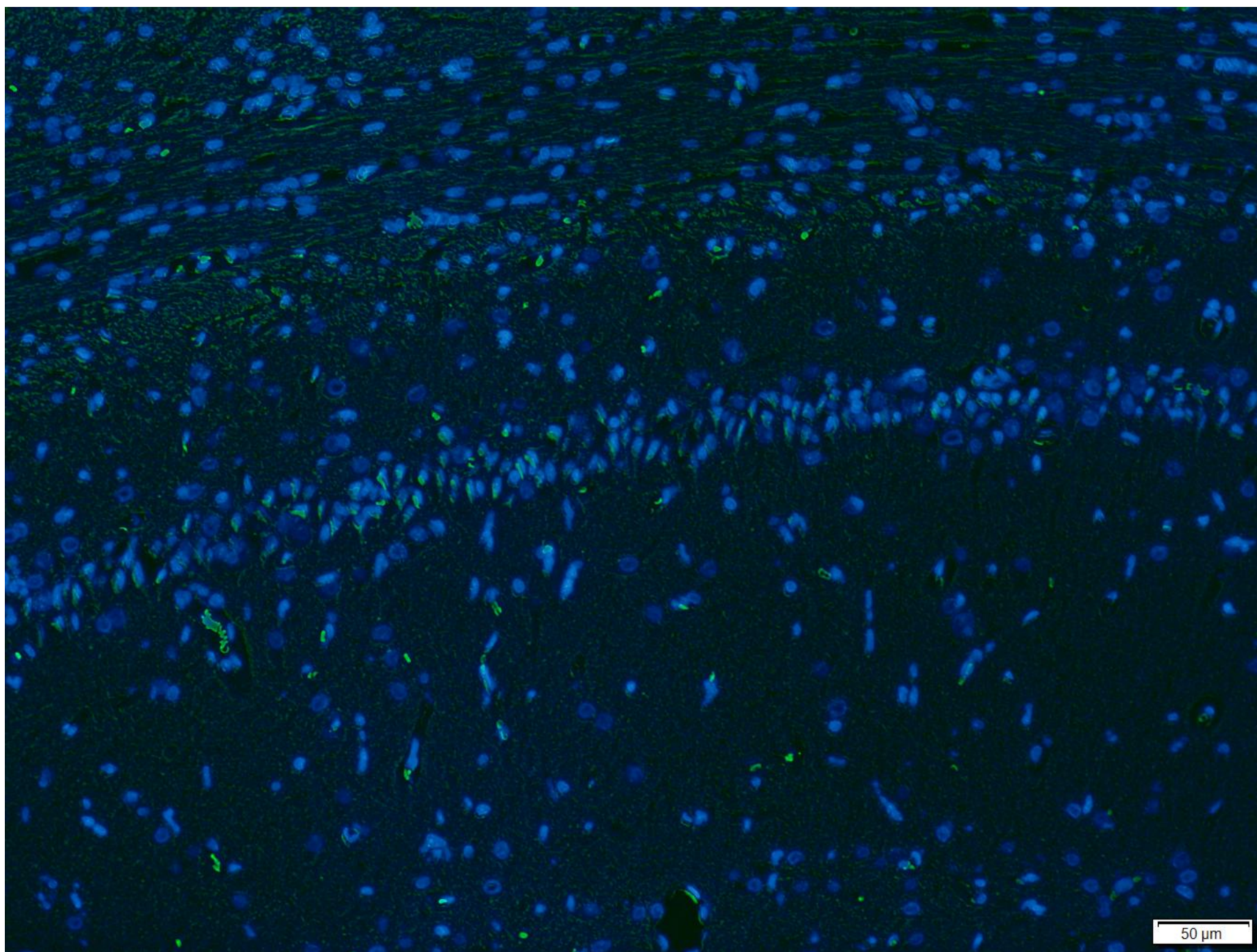

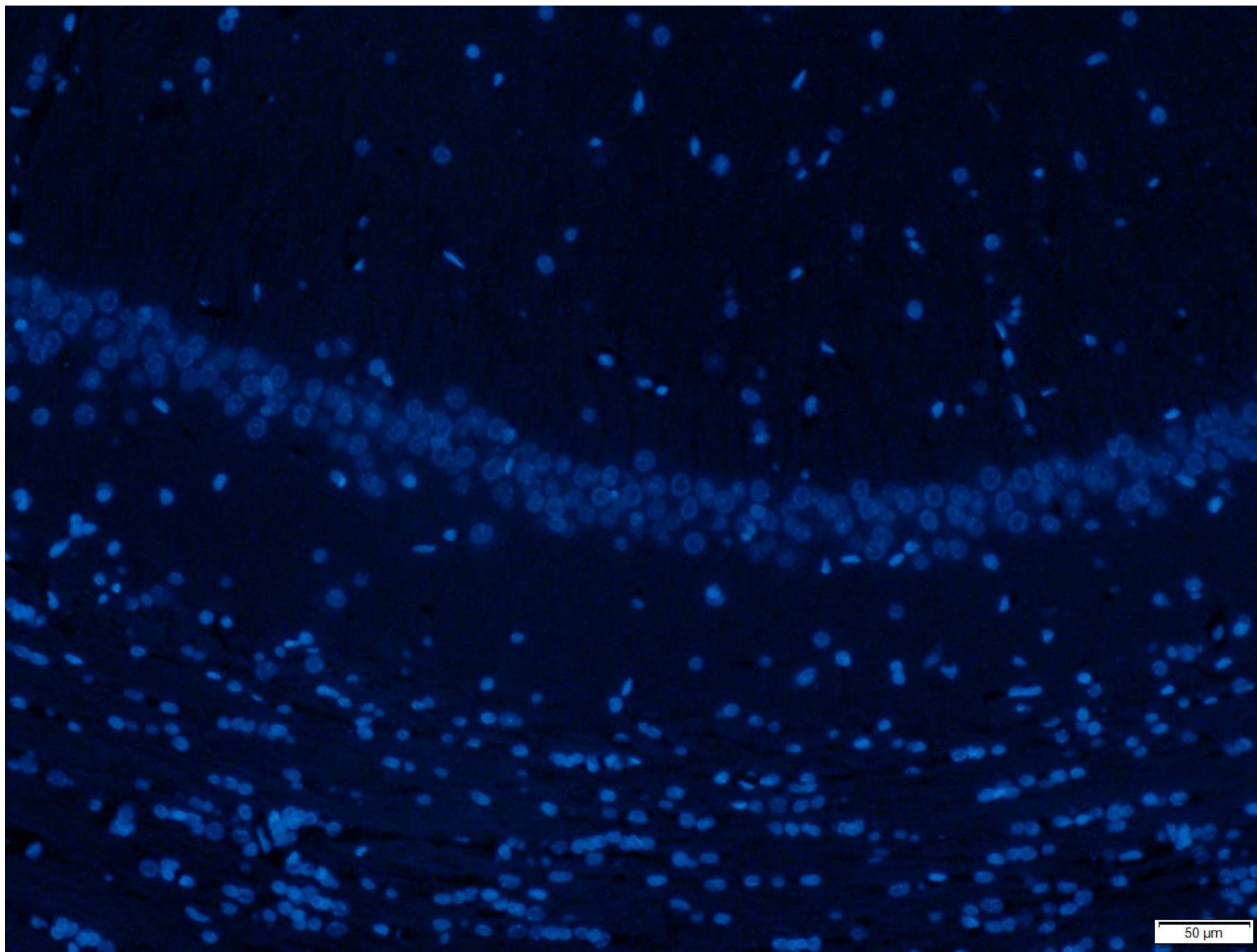

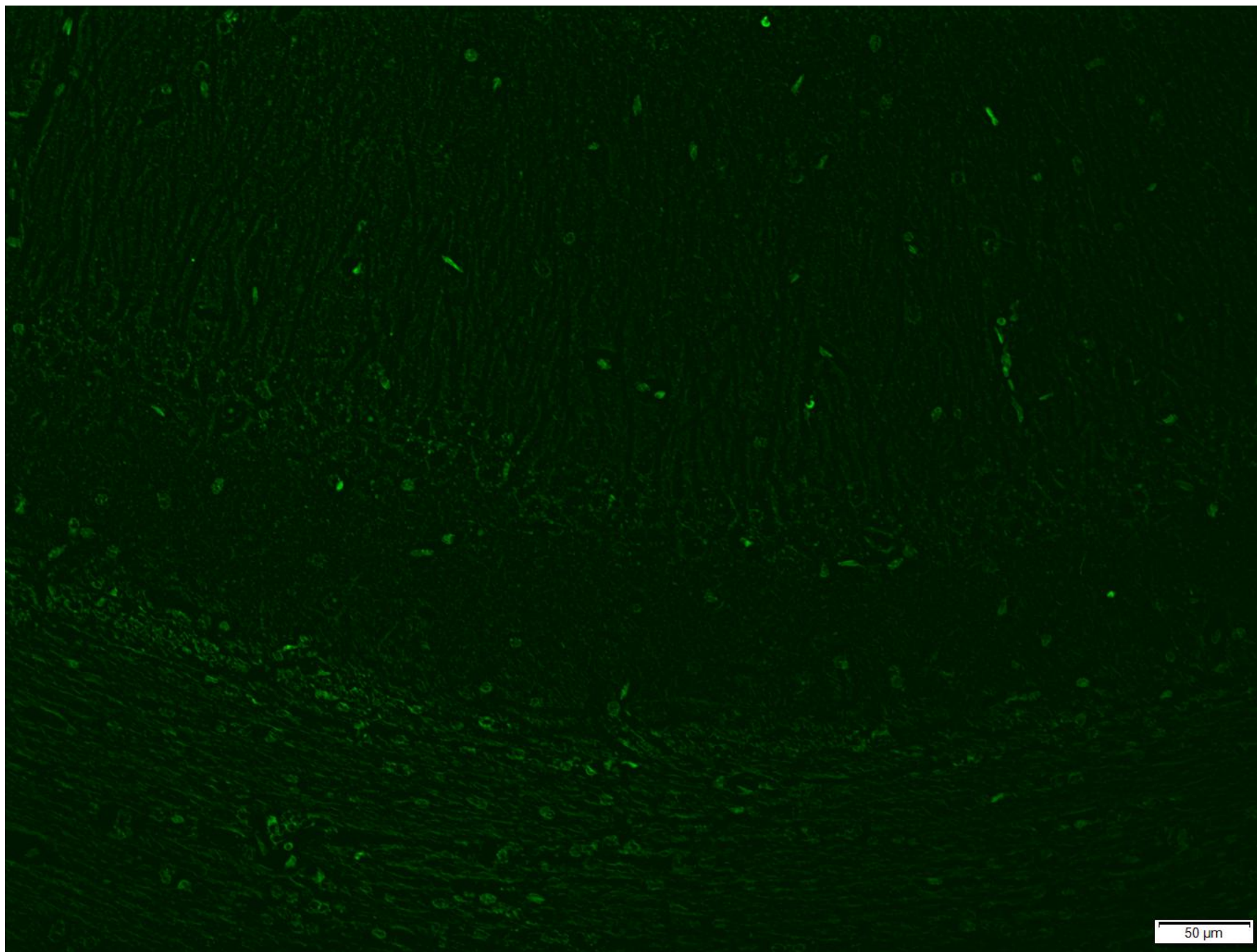

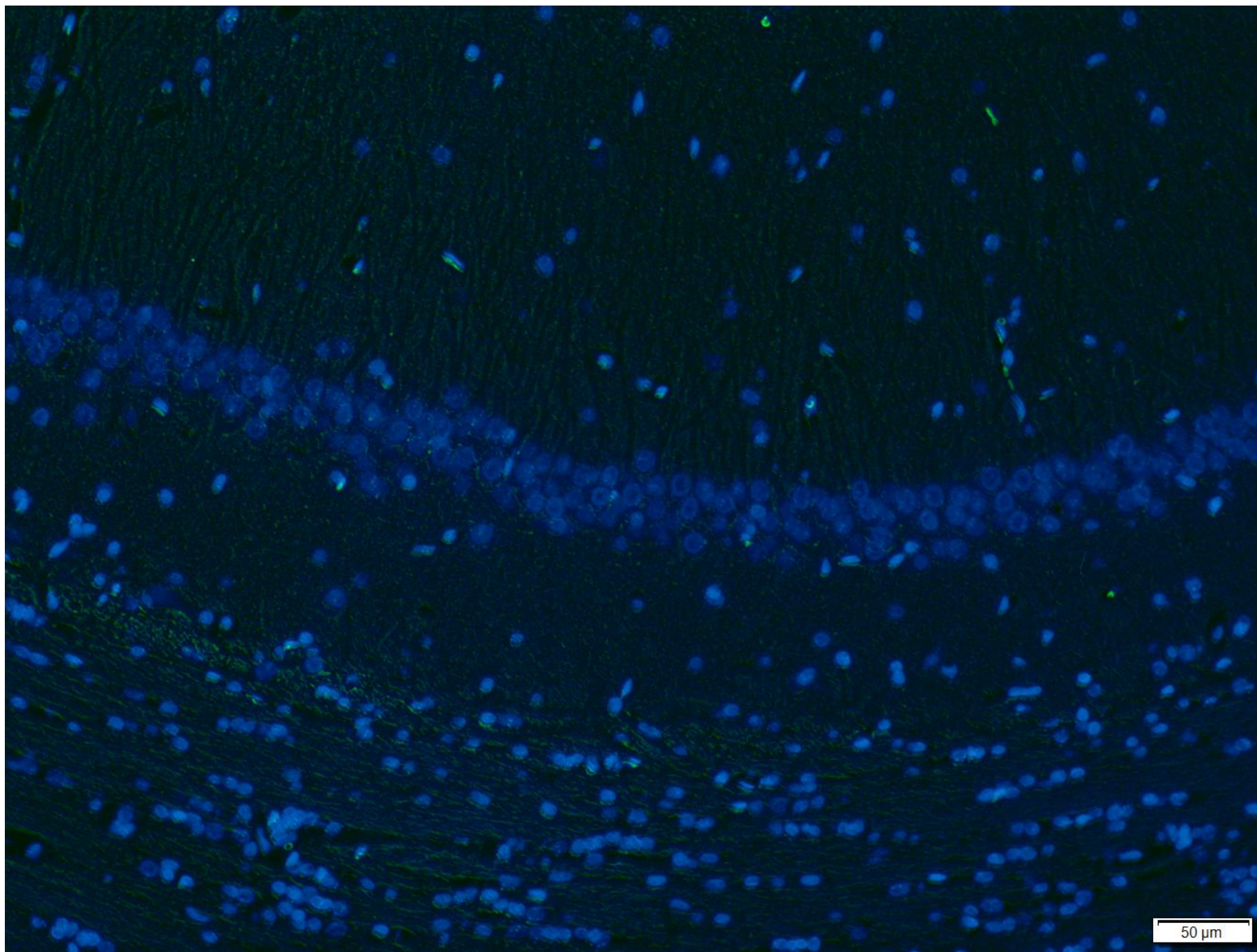

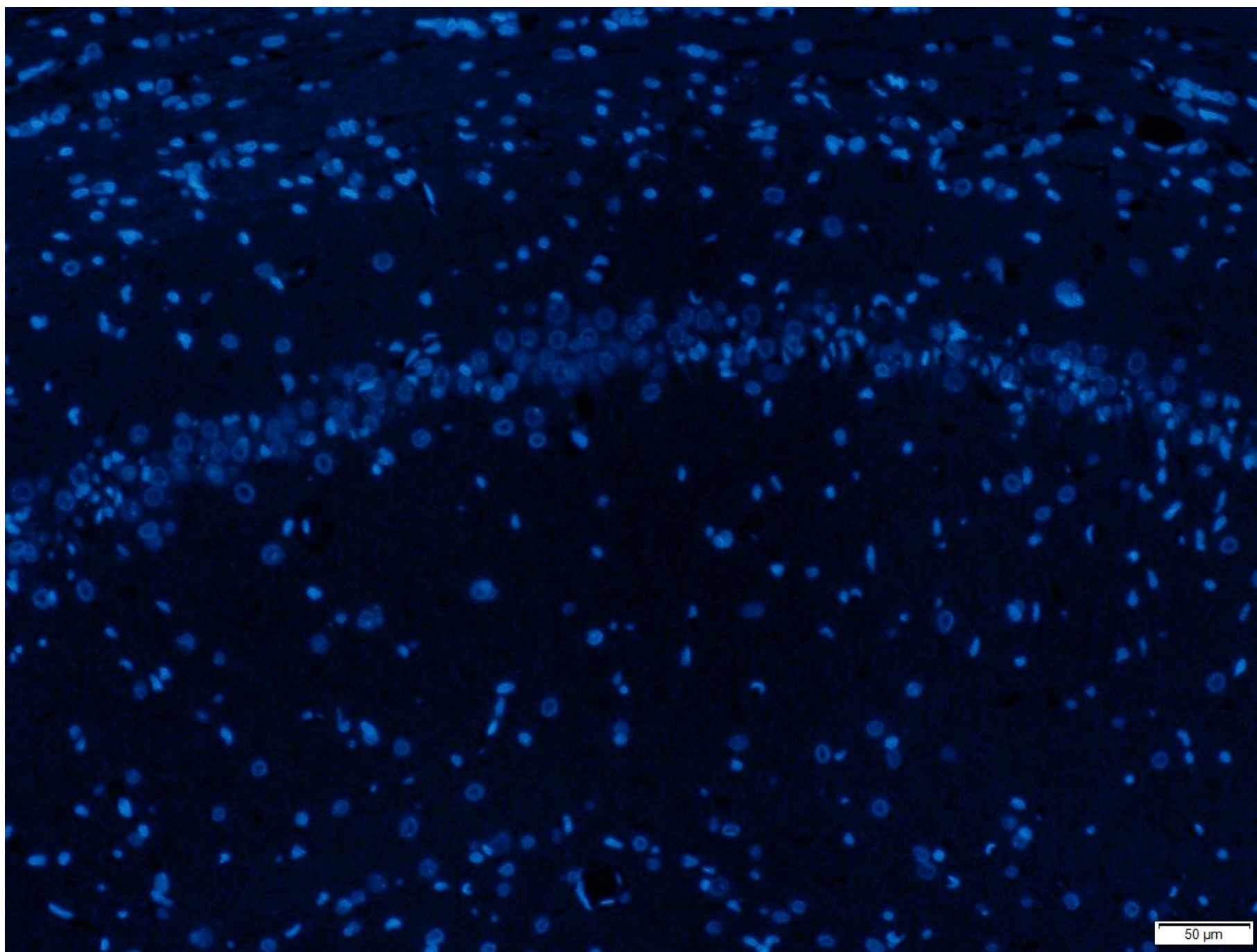

Supplement: S1 Data — (ZIP) [file pone.0339455.s004.zip › TUNEL.pdf]

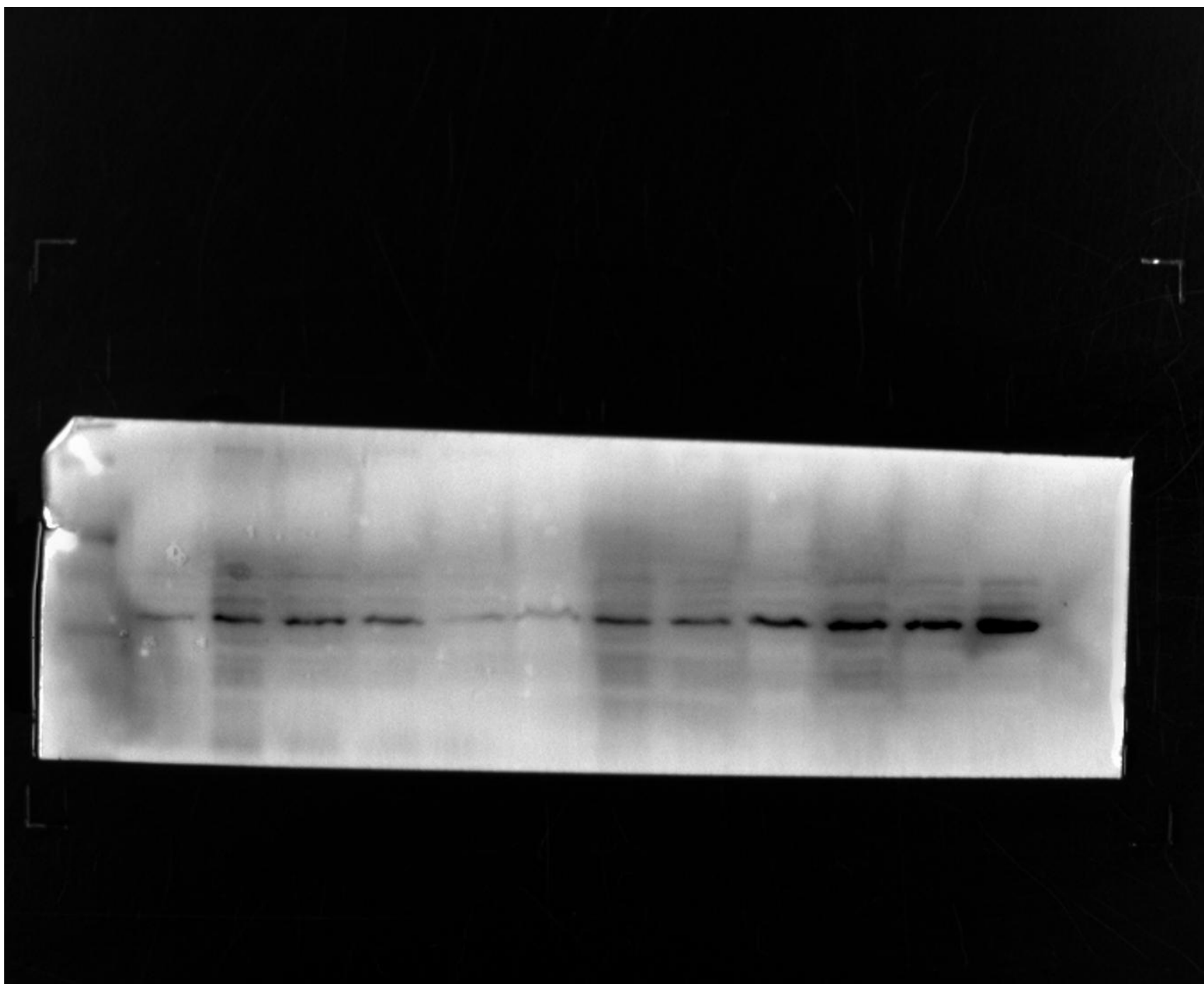

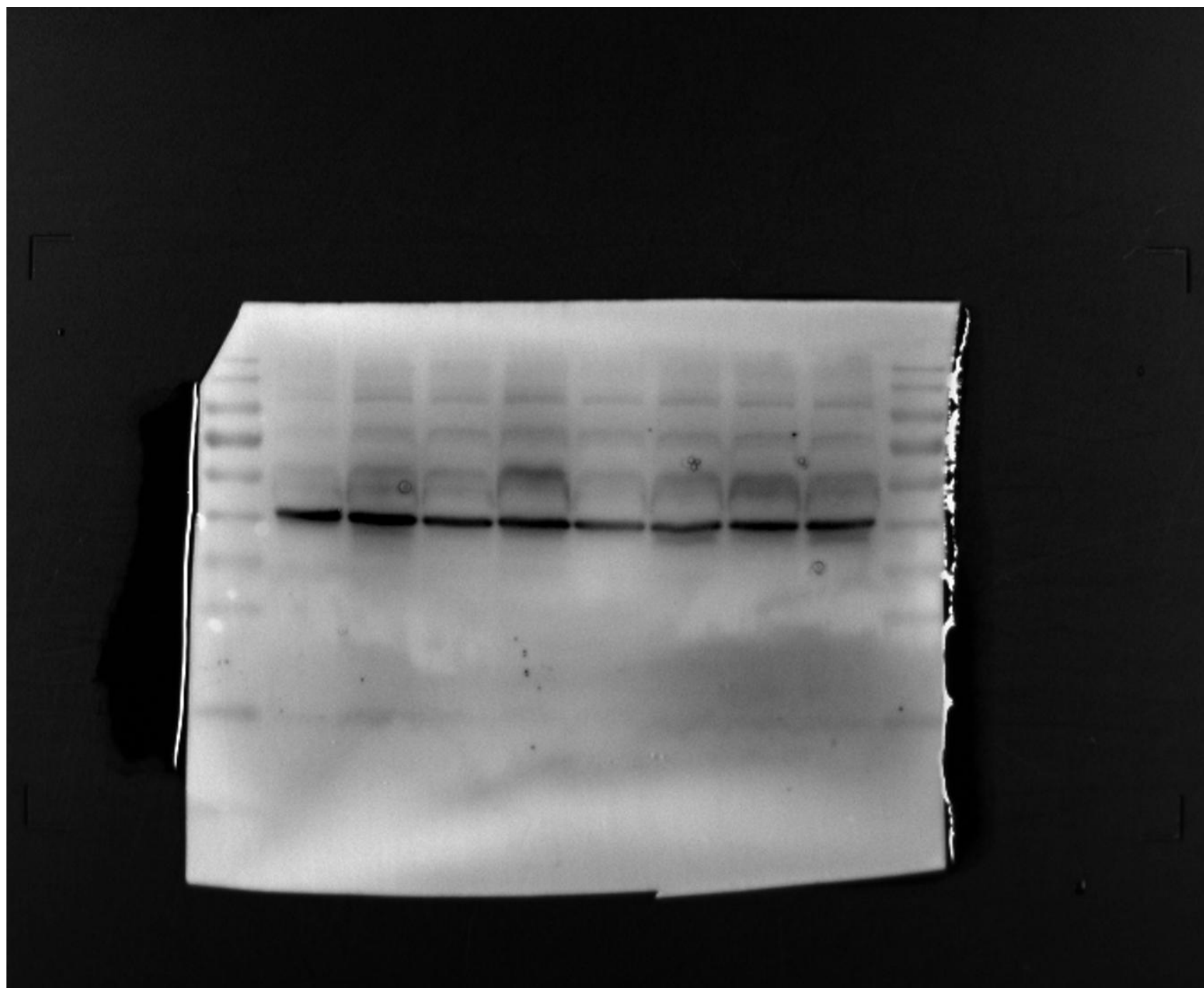

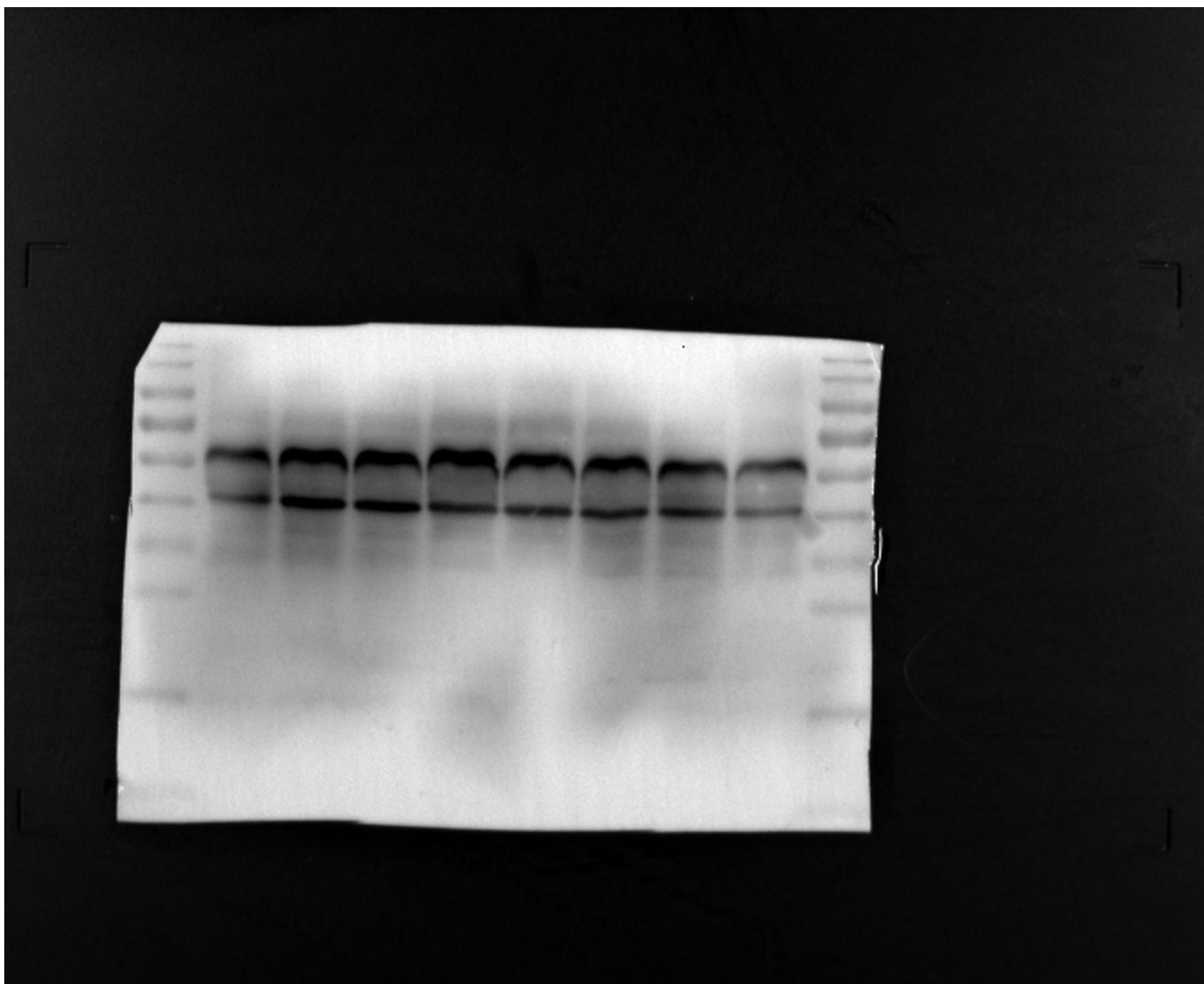

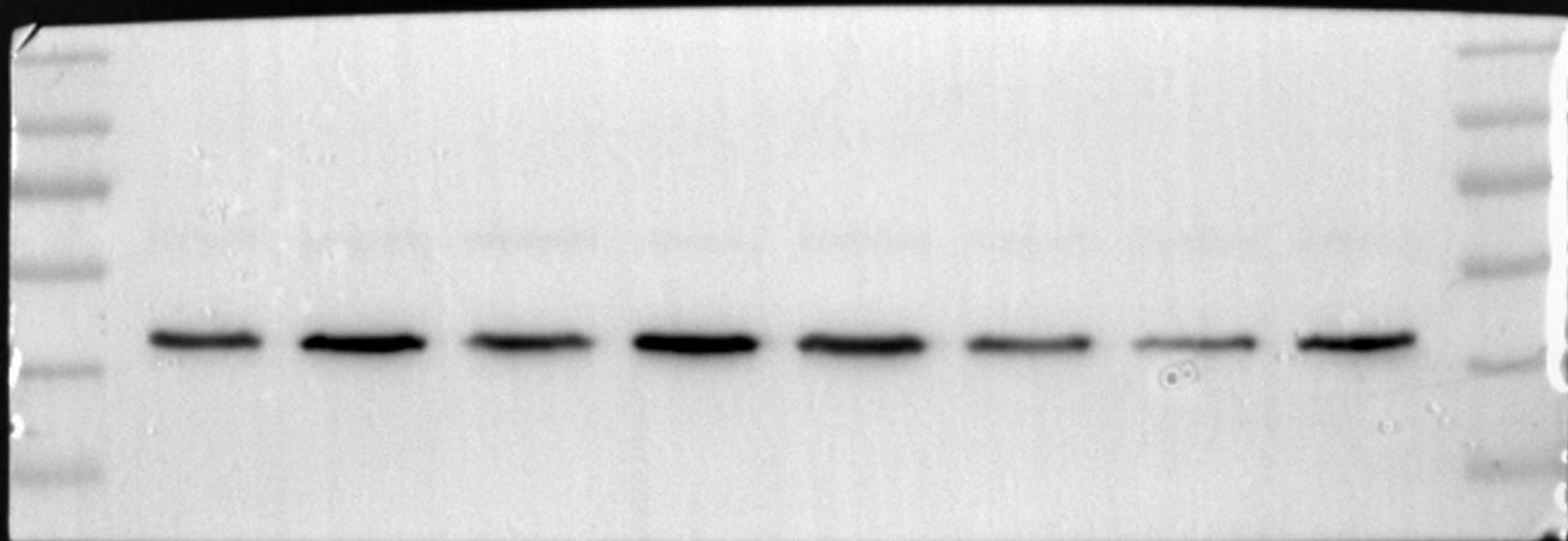

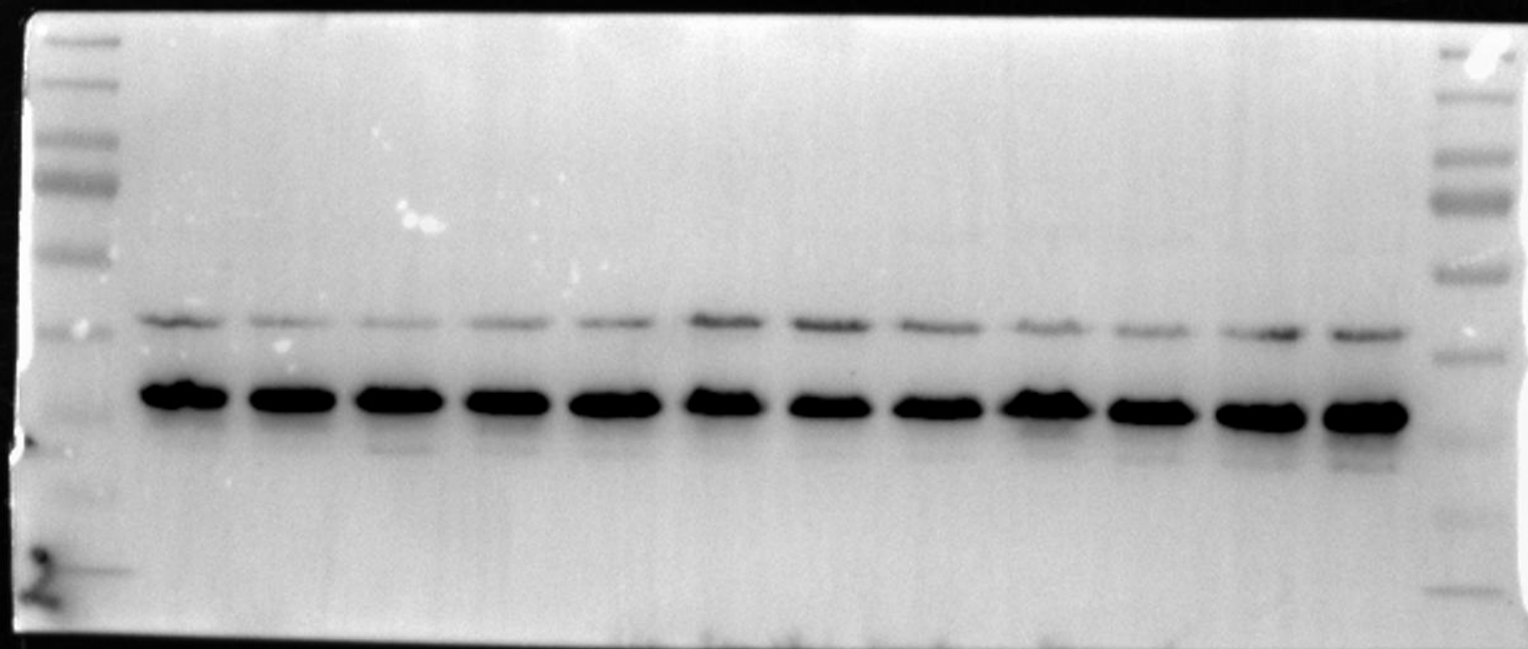

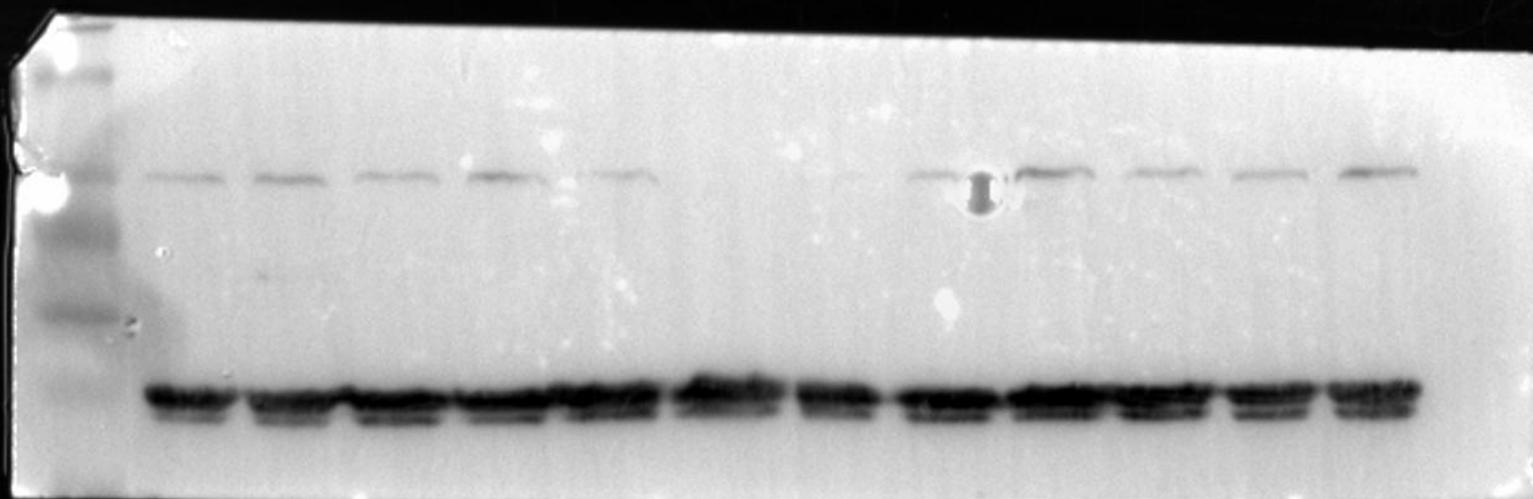

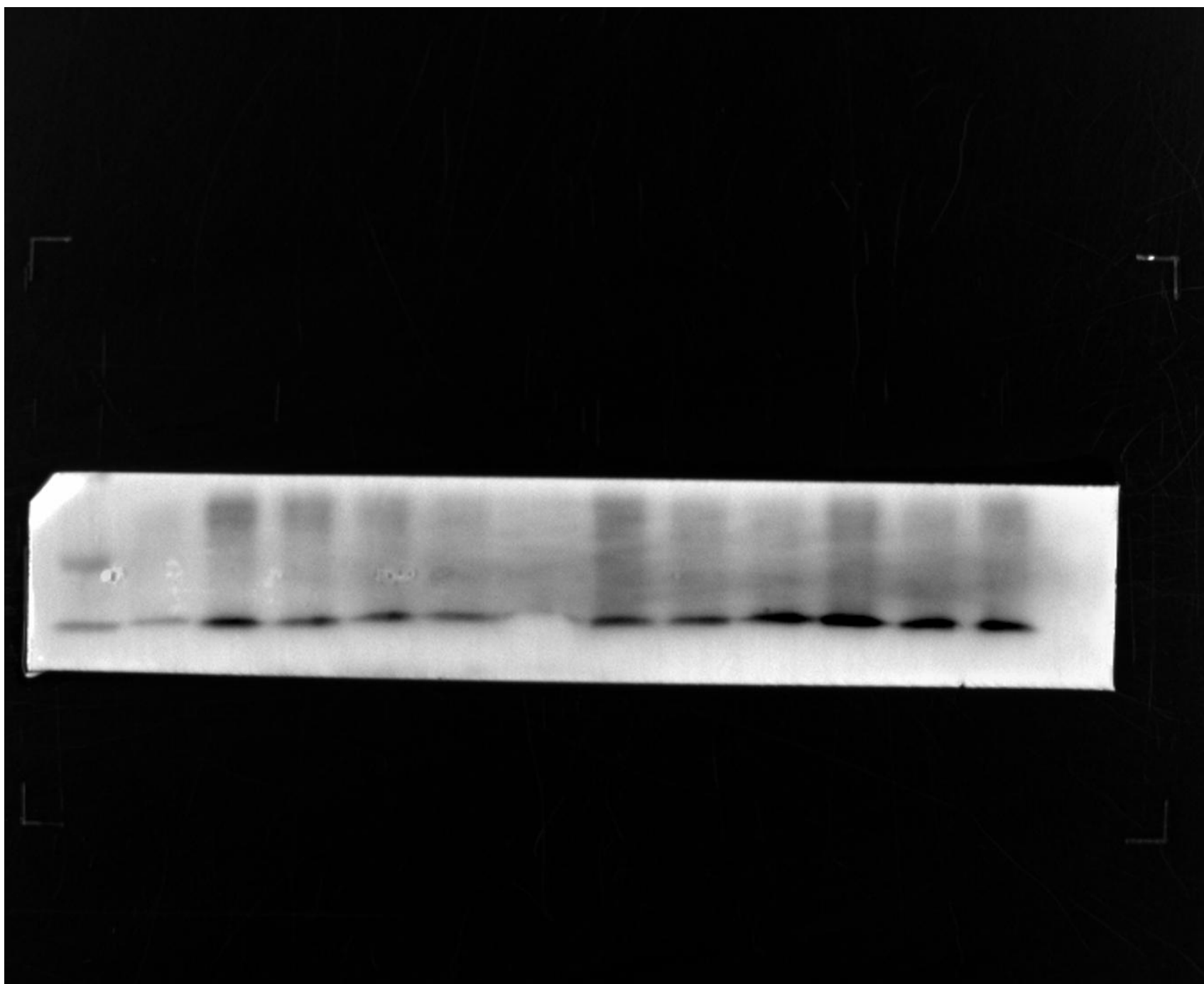

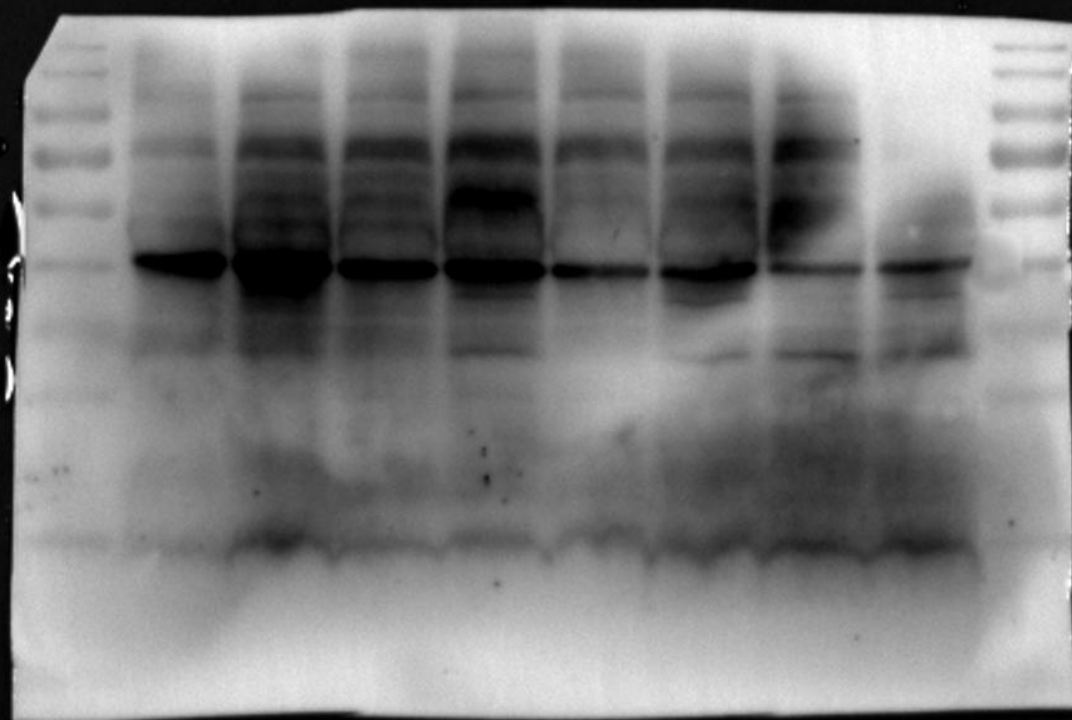

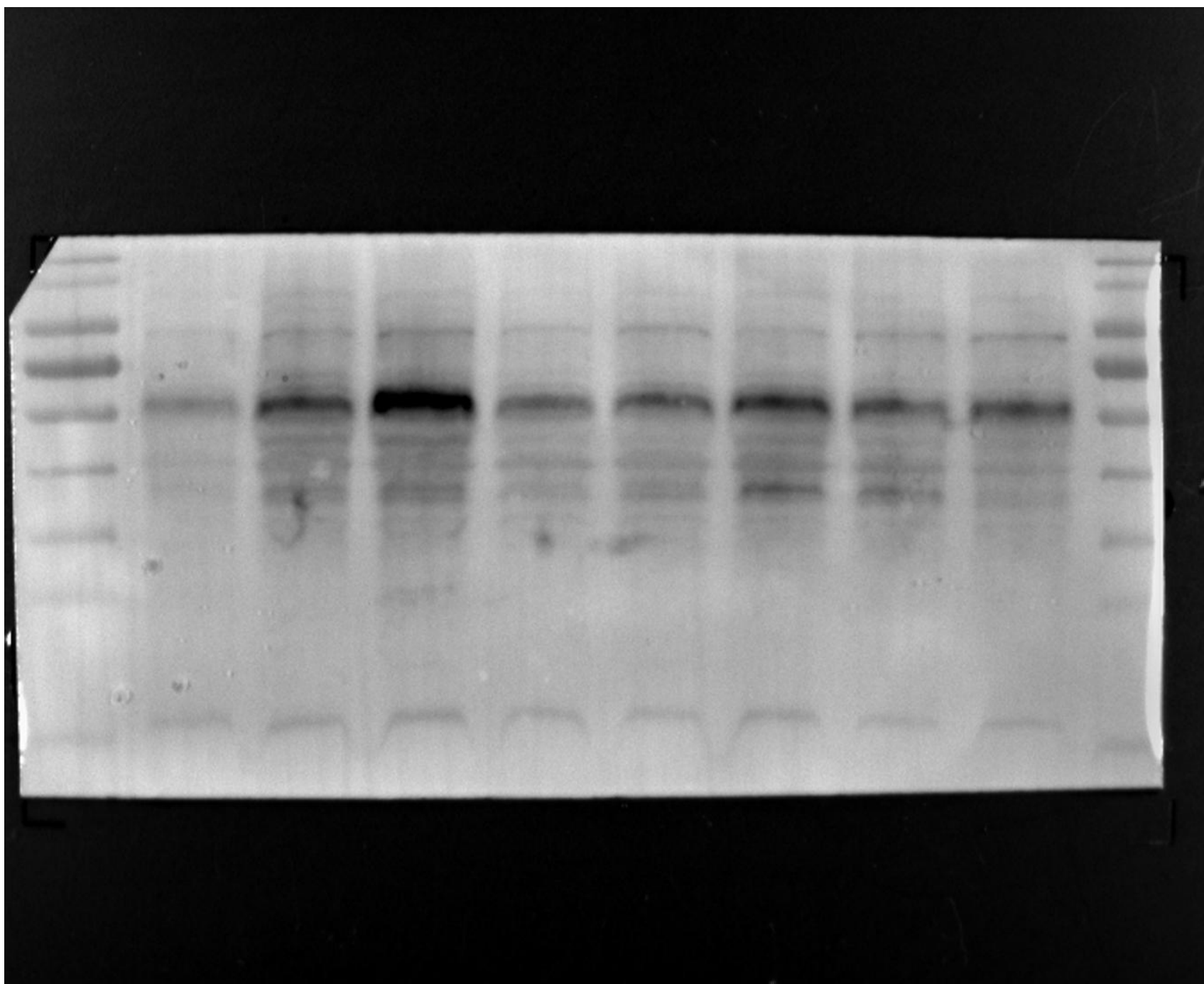

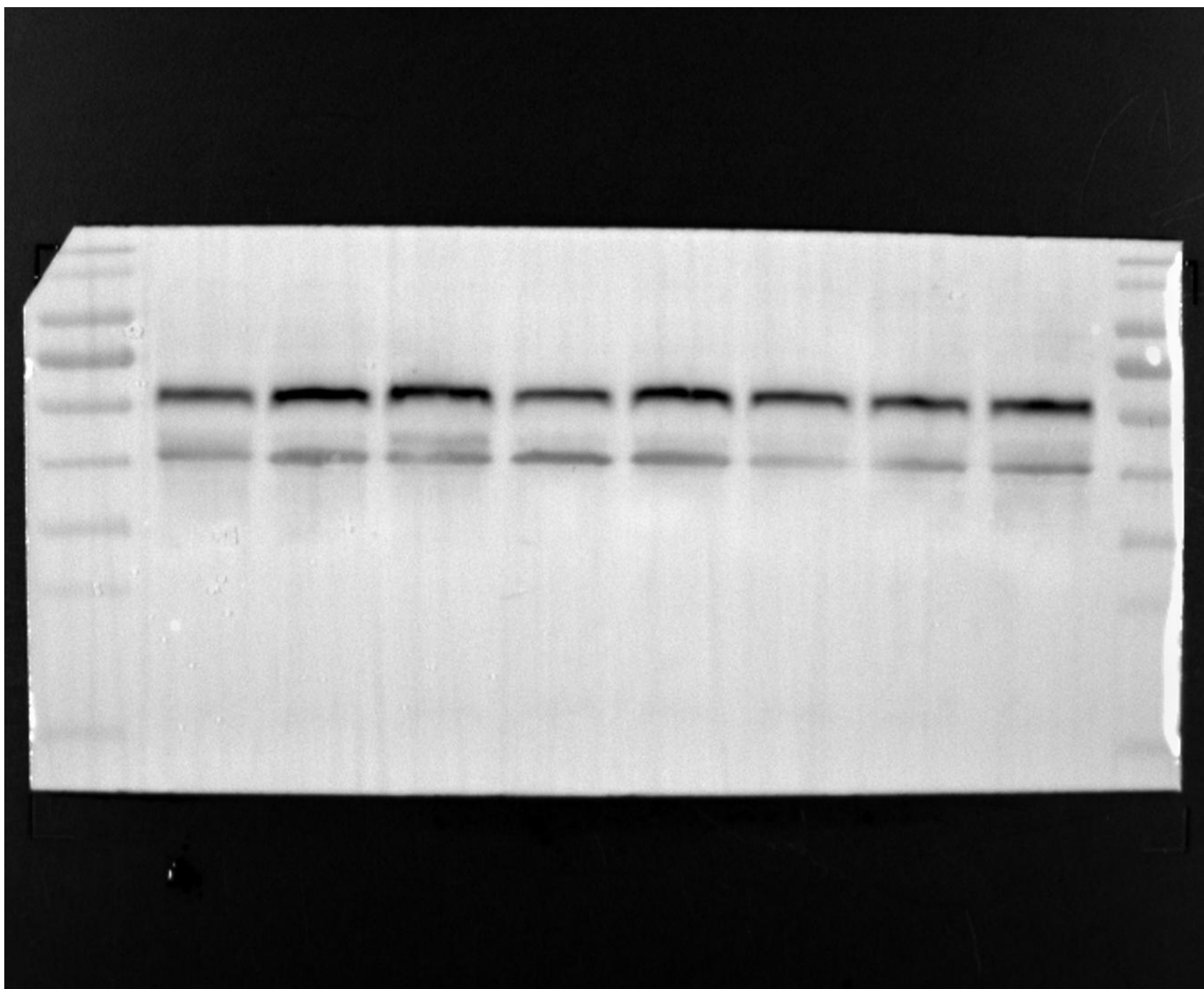

Supplement: S1 Data — (ZIP) [file pone.0339455.s004.zip › WB.pdf]
